# Isocyanides Versus Nitriles: Divergent Hydrogen Bonding Behavior Driven by the Balance Between Dispersive and Electrostatic Forces

**DOI:** 10.1002/cphc.202500834

**Published:** 2026-03-13

**Authors:** Alexander Kanzow, Martin A. Suhm, Margarethe Bödecker

**Affiliations:** ^1^ Institut für Physikalische Chemie Georg‐August‐Universität Göttingen Tammannstr. 6 37077 Göttingen Germany

**Keywords:** hydrogen bonds, intermolecular balances, IR spectroscopy, solvation, supersonic jet

## Abstract

Isocyanides as hydrogen‐bond acceptors are characterized using jet‐cooled Fourier transform infrared spectroscopy for the first time. The hydrogen‐bonded structures of *tert*‐butyl isocyanide (*t*‐BuNC) and its constitutional isomer pivalonitrile (*t*‐BuCN) with a single H_2_O or *tert*‐butyl alcohol (*t*‐BuOH) molecule are analyzed. The most stable monohydrate structures differ markedly: *t*‐BuNC adopts a classical *σ*‐type hydrogen bond, whereas *t*‐BuCN favors a dispersion‐stabilized orthogonal *π*‐type arrangement. Substitution of H_2_O with the more polarizable *t*‐BuOH enhances dispersion interaction between the molecules and drives both complexes toward *π*‐type binding motifs. These findings highlight the balance between dispersive and electrostatic interactions in governing noncovalent binding preferences.

## Graphical abstract

While isocyanides favor *σ*‐type hydrogen bonding, as illustrated by *t*‐BuNC···H_2_O (top), nitriles prefer *π*‐type hydrogen bonding, as illustrated by *t*‐BuCN···H_2_O (bottom). The *σ*‐type complex is characterized by a larger OH vibrational downshift due to more efficient charge transfer, whereas the *π*‐type complex is mainly stabilized by dispersion and thus exhibits a smaller OH vibrational downshift.

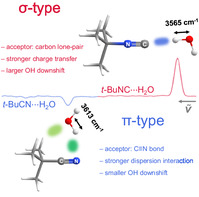

1

Isocyanides (R—N≡C) are versatile, naturally occurring^[^ ^1^ ^]^ building blocks in modern organic synthesis, renowned for their distinct reactivity in multicomponent reactions^[^ ^2^ ^]^ and transition‐metal catalysis.^[^ ^3^ ^]^ While their covalent chemistry is well characterized,^[^ ^4^ ^]^ their noncovalent interactions including hydrogen bonding remain underexplored. The constitutionally isomeric nitriles (R—C≡N), such as the popular solvent acetonitrile, are considerably less reactive^[^ ^4^ ^]^ than their formally isoelectronic^[^ ^5^ ^]^ isocyanide counterparts.

Historically, hydrogen‐bond acceptors were conceived primarily as highly electronegative atoms (F, O, and N).^[^ ^6^ ^]^ However, modern definitions of the hydrogen bond have broadened this conception to include any atom more electronegative than hydrogen and even *π*‐systems.^[^ ^7^ ^]^ In this light, the isocyanide/nitrile isomerism provides an instructive modulation of the terminal lone‐pair *σ*‐acceptor sites and the *π*‐accepting capabilities of the electron‐rich C≡N bonds.

Both *σ*‐^[^ ^8^ ^]^ and *π*‐acceptor^[^ ^9^, ^10^ ^]^ structures of hydrogen‐bonded microsolvates have been computationally identified as local minima in small model systems. This suggests that experimental detection of both motifs for a given donor–acceptor combination is feasible. An investigation of previous gas‐phase microwave studies on hydrogen‐bonded nitriles implies that for the majority, *π*‐bound structures are adopted,^[^ ^11^, ^12^, ^13^, ^14^, ^15^ ^]^ whereas *σ*‐bound structures are rarely found experimentally.^[^ ^15^, ^16^, ^17^ ^]^ Since *tert*‐butylisocyanide (*t*‐BuNC)^[^ ^18^ ^]^ represents the most commonly used isocyanide in literature,^[^ ^19^ ^]^ we focus on this compound and its constitutional isomer pivalonitrile (*t*‐BuCN)^[^ ^20^, ^21^ ^]^ as hydrogen‐bond acceptors. In this study, we pair them with H_2_O or *tert*‐butyl alcohol (*t*‐BuOH) as hydrogen‐bond donors to explore the influence of donor polarizability on the preferred binding site. Using jet‐cooled gas‐phase Fourier transform infrared (FTIR) spectroscopy, we characterize all four donor–acceptor combinations, reporting the first vibrational signatures of hydrogen‐bonded isocyanides under vacuum‐isolation conditions at low temperature.

We support experimental data with quantum‐chemical calculations using ORCA.^[^ ^22^ ^]^ Conformers located with CREST^[^ ^23^, ^24^ ^]^ were optimized at the B3LYP/def2‐TZVP^[^ ^25^, ^26^, ^27^, ^28^, ^29^ ^]^ and B2PLYP/ma‐def2‐QZVP^[^ ^30^, ^31^, ^32^ ^]^ levels of computation, applying three‐body (abc) inclusive D3^[^ ^33^ ^]^ dispersion correction with Becke–Johnson (BJ) damping.^[^ ^34^, ^35^, ^36^, ^37^ ^]^ DLPNO‐CCSD(T)^[^ ^38^, ^39^, ^40^ ^]^ single‐point energies on B2PLYP geometries refined zero‐point corrected conformational energies, constituting the DLPNO‐CCSD(T)//B2PLYP‐D3(BJ,abc)/ma‐def2‐QZVP composite approach (see Section S2, Supporting Information for full computational details).

For all donor–acceptor systems, both *σ*‐ and *π*‐type conformers were found computationally as seen in **Figure** **1**. Notably, computation predicts *σ*‐type coordination to only be preferred for H_2_O···*t*‐BuNC (A) with an almost negligible conformational energy advantage of 0.2 kJ mol^−1^.

**Figure 1:**
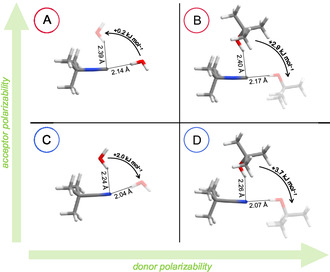
Computed global minimum and lowest energy local minimum structures of A) H_2_O···*t*‐BuNC, B) *t*‐BuOH···*t*‐BuNC, C) H_2_O···*t*‐BuCN, and D) *t*‐BuOH···*t*‐BuCN. Structures were optimized at B2PLYP‐D3(BJ,abc)/ma‐def2‐QZVP level of theory. Hydrogen‐bond donors of the global minimum structures (experimentally observed in all cases) and of the metastable structures (experimentally observed in (B) and (C)) are depicted in opaque and transparent, respectively. Zero‐point corrected energy differences (DLPNO‐CCSD(T)//B2PLYP‐D3(BJ,abc)/ma‐def2‐QZVP) are given in kJ mol^−1^, hydrogen‐bond distances in Å. Room temperature entropy contributions are provided in Table S8, Supporting Information. See also Figures S24–S29, Supporting Information for other illustrations of the bonding situation.

Although the quoted energy differences involve harmonically estimated vibrational zero‐point energy corrections, we expect these corrections to be accurate within about 10% of the total zero‐point correction to the binding energy, the latter being about 6 kJ mol^−1^ for water complexes and about 3 kJ mol^−1^ for *t‐*BuOH complexes (see Table S11, Supporting Information), remarkably independent on *π*‐ or *σ*‐coordination for most isomer pairs.

Increasing donor polarizability (H—OH → *t*‐Bu—OH, B) or swapping the positions of carbon and nitrogen atoms (*t*‐BuNC → *t*‐BuCN, C) is sufficient to invert the conformational preference. When both modifications are combined, the nearly isoenergetic conformers (A) shift to a *π* preference by almost 4 kJ mol^−1^ (D).

Across all systems, *σ*‐type hydrogen bonds are consistently predicted shorter by about 0.2 Å compared to the orthogonal *π*‐type bonds. The longer hydrogen bonds in *t*‐BuNC relative to *t*‐BuCN are consistent with the larger van der Waals radius^[^ ^41^ ^]^ of carbon (1.77 Å) compared to nitrogen (1.66 Å).^[^ ^42^ ^]^ This suggests a uniformly larger steric demand of the isocyanide group, whereas the long‐range electrostatics are similar^[^ ^43^ ^]^ (see also Table S12, Supporting Information).

The apparent N≡C/C≡N homology at short and long range is lost at hydrogen‐bond distance. While similar donor binding energies are predicted for *σ*‐type complexes, *π*‐type complexes profit from about 2 kJ mol^−1^ preference for the nitrile compared to the isocyanide and another 2 kJ mol^−1^ advantage for polarizable donors (see Figure S29, Supporting Information), with the reservation of the limited accuracy of the DLPNO‐CCSD(T) method.

For the equivalent *σ‐*type binding energies of nitriles and isocyanides, molecular orbital (MO) theory may provide a qualitative rationale. Since spatial overlap of the interacting orbitals is expected to be smaller for *t*‐BuNC systems due to larger intermolecular separation, this should translate into weaker interactions. However, this may be compensated through a more favorable donor–acceptor resonance between the isocyanide lone‐pair with the σ*(O—H) donor orbital. This is backed by a comparison of occupied frontier acceptor orbitals as illustrated in **Figure** **2**.

**Figure 2:**
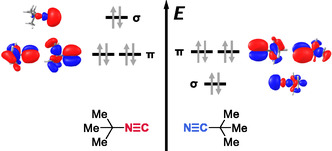
Schematic illustration of the three HOMOs in *t*‐BuNC (left) and *t*‐BuCN (right) and their relative energies. Orbital isosurfaces and energies were computed at B3LYP‐D3(BJ,abc)/def2‐TZVP level of theory. The orbitals are labeled *σ*, *π* based on their irreducible representation of the molecular *C*_3v_ point group (*σ* for A_1_ and *π* for E orbitals).

Computations show that constitutional isomerism leaves the energies of the degenerate *π*‐orbitals essentially unchanged. However, for *t*‐BuNC, the more diffuse carbon‐centered *σ*‐orbital becomes the highest occupied molecular orbital (HOMO), whereas, for *t*‐BuCN, the nitrogen‐centered *σ*‐orbital lies below the *π*‐orbitals. This orbital reordering enhances stabilization through increased charge delocalization in *σ*‐coordination for *t*‐BuNC. Natural bond orbital (NBO) analysis^[^ ^44^ ^]^ at the B3LYP‐D3(BJ,abc)/def2‐TZVP level corroborates this picture, predicting nearly twice the (formal)^[^ ^45^ ^]^ charge transfer to σ*(O—H) in *σ*‐type H_2_O···*t*‐BuNC (0.023*e*) compared to *σ*‐type H_2_O···*t‐*BuCN (0.012*e*). The resulting gain in interaction energy outweighs the *π*‐type dispersion advantage, when H_2_O acts as the donor. In all other systems, dispersion interaction between the molecules dominates and *π*‐coordination prevails, highlighting how subtle differences in electronic structure between isocyanides and nitriles translate into divergent hydrogen bonding preferences.

This correlates with the lower solubility of isocyanides in aqueous media, exemplified by the limited solubility of MeNC in H_2_O compared to the complete miscibility of MeCN with H_2_O.^[^ ^46^ ^]^ The larger intermolecular distances in isocyanide hydration are less competitive with the hydrogen‐bond network of water.

Beyond such necessarily vague bulk phase speculations, we turn to direct experimental validation of the subtle computed conformational preferences. To this end, we employed jet‐cooled FTIR spectroscopy, which enables observation of small complexes under vacuum‐isolated, low‐temperature conditions. In this approach, the hydrogen‐bonded O—H stretching vibration (OHb) serves as a sensitive probe of the local environment. The spectra of the four investigated donor–acceptor systems (**Figure** **3**) were taken on the gratin‐jet, a slit‐jet spectrometer with gas‐recycling (see the Sections 1.2 and 1.3, Supporting Information for full experimental details).^[^ ^47^, ^48^, ^49^ ^]^

**Figure 3:**
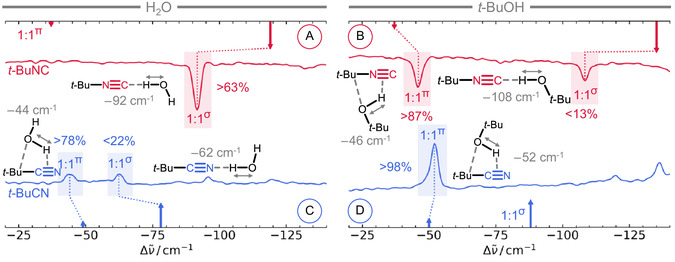
Jet‐cooled FTIR spectra of *t*‐BuNC with A) H_2_O or B) *t*‐BuOH, and of *t*‐BuCN with C) H_2_O or D) *t*‐BuOH, plotted as wavenumber shifts from the monomer band at 3657 cm^−1^ (H_2_O) or 3642 cm^−1^ (*t*‐BuOH). All measurements were performed with a partial pressure of 0.4 mbar (H_2_O, *t*‐BuOH) or 0.2 mbar (*t*‐BuCN, *t*‐BuNC) in 400 mbar He. If possible, harmonic wavenumber shifts (arrows, B3LYP‐D3(BJ,abc)/def2‐TZVP, relative to computation, see Table S10, Supporting Information) of the 1:1‐complexes are assigned to experimental bands. A comparison to absolute wavenumbers is illustrated in Figure S30, Supporting Information. The arrow size represents the integrated absorption coefficient. The relative abundance (%) of the two possible conformations is deduced from experimental band integrals and computed intensities, assuming absence of spectral overlap (Figure S4, Table S13, Supporting Information). For system (A), only the *σ*‐type complex is observed, whereas both the *σ* and *π*‐type structures appear in spectra (B) and (C). For (D), only the *π*‐type structure is detected.

Cluster stoichiometries were established by varying analyte concentration (see Figures S1–S10, Supporting Information for the complete set of spectra). The assigned OHb bands of the 1:1 complexes are all located in the 3620–3530 cm^−1^ wavenumber region (**Table** **1**). They are spectrally as narrow as one would expect for weakly bound complexes, although a recent vacuum ultraviolet‐infrared study suggests otherwise.^[^ ^50^ ^]^ For H_2_O···*t*‐BuNC (A), one distinct signal at 3565 cm^−1^ is observed, corresponding to a 92 cm^−1^ downshift from the symmetric H_2_O monomer stretching fundamental (3657 cm^−1^).^[^ ^51^ ^]^ This agrees much better with the harmonic downshift prediction for the global minimum structure (*σ*‐type) of 119 cm^−1^ than for the almost isoenergetic *π*‐type structure of 37 cm^−1^. With this assignment, the coexistence of the *π*‐type conformer would remain undetectable at populations up to 36% at the available signal‐to‐noise ratio, owing to the small predicted integrated absorption coefficient of the *π*‐type conformer (≈12% of the *σ* integrated absorption coefficient, Table S8, Supporting Information).

**Table 1:** Experimentally determined OHb wavenumbers ${\tilde{\nu }}_{\text{exp}}$ and downshifts $-\Delta \tilde{\nu }={\tilde{\nu }}_{\text{mono}}-{\tilde{\nu }}_{\text{dim}}$ of assigned dimer bands from the monomer band together with the observed signal intensity $I_{\text{exp}}$, determined from the spectra shown in Figure 3.

	${\tilde{\nu }}_{\text{exp}}\;/\;{\text{cm}}^{-1}$	$-\Delta \tilde{\nu }\;/\;{\text{cm}}^{-1}$	$I_{\text{exp}}\;/\;\mu m^{-1}$	Assignment
A	3565(1)	92(1)	3.38(22)	1:1^*σ*^
B	3534(1)	108(1)	1.53(22)	1:1^*σ*^
	3596(1)	46(1)	2.55(22)	1:1^*π*^
C	3595(1)	62(1)	0.67(22)	1:1^*σ*^
	3613(1)	44(1)	0.86(22)	1:1^*π*^
D	3590(1)	52(1)	4.15(22)	1:1^*π*^

In the FTIR spectrum of *t*‐BuOH···*t*‐BuNC (B), two 1:1 complex signals, separated by 62 cm^−1^, are observed. According to harmonic predictions, the higher wavenumber band at 3596 cm^−1^ (46 cm^−1^ downshift) is to be assigned to the energetically favored *π*‐type conformer. Despite the energetic disadvantage of 2.9 kJ mol^−1^, the *σ*‐type complex is still observed in the spectrum (3535 cm^−1^, 108 cm^−1^ downshift). This can be explained by the approximately fivefold higher integrated absorption coefficient of the metastable conformer (Table S8, Supporting Information), which allows for its detection even at low abundances.

Both dimer structure types are also observed for H_2_O···*t*‐BuCN (C). Analogously to B, the bands at 3613 cm^−1^ (44 cm^−1^ downshift) and 3595 cm^−1^ (62 cm^−1^ downshift) can be assigned to the energetically favored *π*‐type structure and the metastable *σ*‐type structure, respectively. Once again, the energetic disadvantage of the *σ*‐complex is compensated by the larger absorption cross‐section.

Experimental confirmation of the metastability of the *σ*‐type conformers in B and C is obtained by employing Ne instead of He as the carrier gas for enhanced collisional cooling.^[^ ^52^ ^]^ Under these conditions, all dimers relax into the global *π*‐type minima (Figures S2 and S3, Supporting Information).

In *t*‐BuOH···*t*‐BuCN (D), the *π*‐type conformer is assigned to the band at 3590 cm^−1^ (52 cm^−1^ downshift). It becomes evident that for all four systems, the deviation between theory and experiment is larger for the classical *σ*‐type than for the *π*‐type hydrogen bond. This observation is consistent with previous studies noting that there is a systematic discrepancy in harmonic B3LYP performance between *π*‐ and *σ*‐type hydrogen‐bond complexation shifts.^[^ ^53^, ^54^, ^55^, ^56^ ^]^

If scaled absolute values rather than wavenumber shifts are considered (Table S8, Figure S30, Supporting Information), it becomes evident that the empirical scaling factor of 0.97 works well for the monohydrate species, whereas the absolute value predictions deviate more from the experimental wavenumbers for the *t*‐BuOH adducts and would require a smaller scaling factor.

From a comparison with the *t*‐BuOH···*t*‐BuNC (B) and H_2_O···*t*‐BuCN (C) combinations, it appears that scaled computation overestimates the *π*‐type wavenumbers for both *t*‐BuNC and *t*‐BuCN. Consequently, the experimental feature in D is better assigned to the *π*‐type conformer, despite the nominally better agreement with the *σ*‐type prediction (see Figure S30, Supporting Information). Additionally, the strongest *π*‐type preference is found for D, which explains why no second conformer can be observed. This might also be a result of spectral overlap, although this is rendered improbable through a comparison to the other systems and harmonic predictions.

Relative populations estimated from the absorbance integrals using integrated absorption coefficients show a trend consistent with computed energy differences as summarized in Figure 1. This consistency supports the band assignments based on comparison with scaled computed harmonic wavenumbers once again. One can turn this argument around and ask when a systematic over‐ or underestimation of *π*‐complex stability by the harmonically corrected CCSD(T) would start to be in conflict with experiment. This is perhaps best seen in system C. If the *π*‐complex energy advantage were off the computed value of 2.0 kJ mol^−1^ by 1.5 kJ mol^−1^ in either direction, the conformational distribution would correspond to an effective Boltzmann population ratio of the two isomers which is far too high or too low to be realistic.

Reflecting the stronger electrostatic contribution in these structures, *σ*‐type coordination induces a more pronounced downshift of the OHb compared to *π*‐type binding. Notably, the *σ*‐type conformers of H_2_O (A, C) are experimentally found to be downshifted by 30 cm^−1^ when *t*‐BuNC is used as the acceptor instead of *t*‐BuCN. This might seem counterintuitive, given that the hydrogen bond in H_2_O···*t*‐BuNC complex is about 0.1 Å longer. However, this trend is well documented in liquid‐phase IR spectra,^[^ ^57^, ^58^ ^]^ rather well replicated by B3LYP‐D3(BJ,abc)/def2‐TZVP computations in this work, and already qualitatively reproduced at the HF/def2‐TZVP level. The similar leading multipole moments do not provide a satisfactory long‐range explanation. In contrast, the MO approach offers an intuitive rationale: Compared to *t*‐BuCN, the enhanced formal charge transfer from *t*‐BuNC into the σ*(O—H) acceptor orbital leads to a more pronounced weakening of the O—H bond as supported by the NBO analysis.

Having addressed the OHb vibrations of *σ*‐type, we now turn to those of *π*‐type. Comparison of B and D reveals that *t*‐BuNC and *t*‐BuCN induce similar downshifts of the OHb band, with *t*‐BuOH···*t*‐BuCN exhibiting a slightly larger shift by 6 cm^−1^. Although subtle, this trend aligns with the slightly higher nitrile *π*‐MO energy. It supports the notion that the key electronic distinction between isocyanides and nitriles lies in the *σ* lone‐pair orbital, while *π*‐orbitals remain largely comparable (Figure 2).

While the OHb vibration is our main experimental probe, the C≡N vibration is more commonly investigated in protein tagging^[^ ^59^, ^60^ ^]^ and as a probe of local electric fields via the vibrational Stark effect.^[^ ^61^, ^62^ ^]^ In the C≡N stretching region, both microsolvation and microcondensation effects can be monitored (**Figure** **4**). In a previous IR spectroscopic study on alcohol−nitrile clusters in CCl_4_ solution,^[^ ^63^ ^]^ an approximately linear correlation between the OHb downshift and C≡N upshift upon hydrogen‐bond formation was observed. Notably, the OHb downshift is about 20 times larger in magnitude than the corresponding C≡N upshift. Moreover, unlike the OHb mode, the C≡N vibration does not exhibit such a significant intensity enhancement upon hydrogen‐bond formation. While, according to harmonic calculations, the nitrile C≡N vibrations experience an approx. twofold increase in absorption coefficient (as reported before in Ref. [64]), the isocyanide N≡C‐stretching absorption coefficient decreases upon hydrogen‐bond formation (Table S9, Supporting Information). These findings underline that, while the C≡N stretch provides valuable complementary information, the OHb vibration remains the far more sensitive probe for hydrogen‐bonding interactions, also in terms of *σ*‐ and *π*‐complex differentiation.

**Figure 4:**
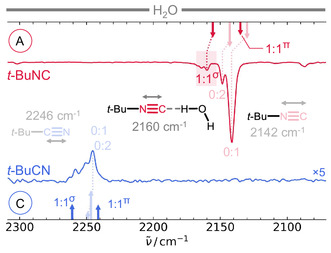
C≡N stretching region of the jet FTIR spectra of A) *t*‐BuNC and of C) *t*‐BuCN (amplified by ×5) with water. Measurements were performed with a partial pressure of 0.4/0.2 mbar (H_2_O/*t*‐BuNC, (A)) or 0.8/0.4 (H_2_O/*t*‐BuCN, (C)) in 400 mbar Ne. For *t*‐BuNC, the homo‐ (0:2) and heterodimer (1:1) N≡C‐stretching band shift to higher wavenumbers compared to the monomer (0:1) band, whereas monomer and homodimer bands coincide for *t*‐BuCN. Scaled harmonic predictions (arrows, Table S9, Supporting Information) at the B3LYP‐D3(BJ,abc)/def2‐TZVP level (scaling factor 0.96) predict downshifts for the *π*‐type heterodimers which are not identified in the spectra. Bands of larger clusters are expected to appear upshifted relative to the monomer bands for both compounds. See Table S6, Supporting Information for experimental wavenumbers and Section 2.5.2, Supporting Information for details on the band integration.

The overlapping bands of the jet‐cooled *t*‐BuCN monomer and homodimer around 2246 cm^−1^ are consistent with the room‐temperature gas‐phase C≡N‐wavenumber at 2254 cm^−1^.^[^ ^65^ ^]^ In the liquid phase, this band is observed at 2234.5 cm^−1^,^[^ ^65^ ^]^ indicating a substantial downshift upon condensation. A corresponding effect is expected in the cold gas phase, where the homodimer represents the smallest possible microcondensate. Indeed, a downshift of the C≡N stretching band for the homodimer relative to the monomer is not only predicted by harmonic calculations but also supported by subtraction analysis of the experimental spectra (Figure S8, Supporting Information).

For the 1:1 *σ*‐ and *π*‐type nitrile complexes, harmonic calculations predict an upshift and a downshift of the C≡N stretching frequency relative to the monomer, respectively, highlighting the recently reported geometry dependence of hydrogen‐bonded nitrile vibrations.^[^ ^66^ ^]^ Considering the computed intrinsic intensity ratio between the OHb and C≡N modes (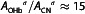; Tables S8 and S9, Supporting Information) and the fact that solely the *π*‐type conformer is observed in the OH region of the FTIR spectrum with Ne as the carrier gas, it is expected that likewise only the *π*‐type complex contributes in the C≡N region. In comparison to the pure pivalonitrile spectrum (Figure S7, Supporting Information), additional weak bands appear in the mixed spectrum. These mixed‐cluster signals cannot be unambiguously assigned to specific cluster stoichiometries due to spectral overlap and seem to appear predominantly at higher wavenumbers than the monomer band, apparently inconsistent with the predicted downshift of the *π*‐type C≡N stretching vibration. However, subtraction analysis suggests that a weak underlying downshift consistent with the harmonic prediction of the *π*‐type complex cannot be excluded (Figure S10, Supporting Information).

The situation is more straightforward for *t*‐BuNC: In the cold gas‐phase spectrum (Figure 4), the IR‐active N≡C‐stretching band of the homodimer appears at 2148 cm^−1^, upshifted by 7 cm^−^^1^ relative to the monomer fundamental. This behavior parallels the previously reported upshift upon condensation of *t*‐BuNC at room temperature (2134 cm^−^^1^ in the gas phase^[^ ^67^ ^]^ vs. 2137 cm^−^^1^ in the bulk phase).^[^ ^68^ ^]^ We find that the N≡C‐stretch is about an order of magnitude more intense in the IR than the C≡N‐stretch, a result previously reported^[^ ^60^ ^]^ and rationalized by the steeper slope of the harmonic N≡C dipole curve (Figure S31, Supporting Information).

Furthermore, combining spectral band integrals with computed intensities reveals that less than 10% of the *t*‐BuNC molecules pair up to form homodimers in the He expansion, while this share increases to about 20% with Ne as the carrier gas (Table S14, Supporting Information).

Two weak additional features are observed at 2087 and 2108 cm^−1^ (Figure S6, Supporting Information), potentially arising from an anharmonic resonance in the monomer species. The main N≡C band position, however, is not significantly affected by this perturbation, since both features together account for less than 10% of the integrated main band intensity.

In the mixed spectra, a band at 2160 cm^−1^ can be assigned to a H_2_O···*t*‐BuNC complex. This feature is attributed to the *σ*‐type conformer, since this structure is the only structure observed in the OH region. Spectral overlap with larger mixed clusters prevents reliable integration of the 1:1 band. Nevertheless, a conservative estimate of the experimental intensity ratio *I*_NC_^*σ*^/*I*_OHb_^*σ *^= 0.35 ± 0.19 agrees well with the harmonic prediction of 0.27. The N≡C‐upshift for this microsolvated system mirrors the trend found for macrosolvated *t*‐BuNC in solution, where solvent‐induced upshifts up to 10 cm^−1^ have been observed.^[^ ^67^ ^]^

In summary, this study provides the first evidence of single hydrogen‐bond formation to isocyanides under jet‐cooled vacuum‐isolation conditions. Like nitriles, isocyanides can engage in both *σ*‐ and *π*‐type hydrogen bonding. However, their preferred binding motifs with water as the donor diverge due to differences in electronic structure. Computations show that this arises from the interplay between stronger dispersive interactions in nitrile complexes, due to shorter intermolecular distances, and the more effective charge transfer in isocyanide *σ*‐complexes. This trend is observed in the vibrational spectra of monosolvates, where *σ*‐type isocyanide hydrogen bonds exhibit larger OHb downshifts despite longer intermolecular distances. The use of a vacuum‐isolation technique allows for distinction of *σ*‐ and *π*‐type hydrogen bonding, which would be largely washed out in bulk solution. Our results demonstrate how the balance between dispersive and electrostatic forces governs qualitatively different hydrogen bonding preferences, providing insights into solvation and noncovalent interactions of isocyanides and nitriles.

## 3 Conflict of Interest

The authors declare no conflict of interest.

## Supporting information

Supplementary Material

## Data Availability

The data that support the findings of this study are openly available in GRO.data at https://doi.org/10.25625/Q4AFC1.
