# Supplementary material for: Isocyanides Versus Nitriles: Divergent Hydrogen Bonding Behavior Driven by the Balance Between Dispersive and Electrostatic Forces
Source: Chemphyschem. 2026 Mar 13;27(5):e202500834. doi: 10.1002/cphc.202500834 (PMC12985692; doi:10.1002/cphc.202500834)
Supplement: Supplementary file 1 — Supplementary Material [file CPHC-27-e202500834-s001.pdf]

Supporting Information on:

Isocyanides vs Nitriles: Divergent Hydrogen Bonding Behavior Driven by the Balance Between Dispersive and Electrostatic Forces

Alexander Kanzow, Martin A. Suhm, Margarethe Bödecker*

Institute of Physical Chemistry, University of Göttingen,
Tammannstr. 6, 37077 Göttingen, Germany

E-Mail: alexander.kanzow@stud.uni-goettingen.de, msuhm@gwdg.de,
m.boedecker@stud.uni-goettingen.de

Contents

1 Experimental Details **S1**

1.1 Data availability . . . S1

1.2 Instrumentation . . . S1

1.3 Investigated Compounds . . . S2

1.4 FTIR Spectra . . . S3

2 Computational Details **S16**

2.1 Methods . . . S16

2.2 Input Files . . . S17

2.3 Structure Output Data . . . S18

2.4 Computed Properties . . . S23

2.5 Experimental-Theoretical Abundances . . . S25

2.5.1 OH region . . . S25

2.5.2 C≡N region . . . S27

3 Non-Covalent Interaction Plots **S30**

4 Other Figures **S33**

List of Figures

S1 FTIR spectra H_2O + *t*-BuNC . . . S5

S2 FTIR spectra *t*-BuOH + *t*-BuNC . . . S7

S3 FTIR spectra H_2O + *t*-BuCN . . . S8

S4 FTIR spectra *t*-BuOH + *t*-BuCN . . . S9

S5 FTIR spectra H_2O + *t*-BuNC (C≡N region) . . . S10

S6 FTIR spectra H_2O + *t*-BuNC (C≡N region) + subtraction spectra . . . S11

S7 FTIR spectra *t*-BuCN (C≡N region) . . . S12

S8 FTIR spectra *t*-BuCN (C≡N region) + subtraction spectra . . . S13

S9 FTIR spectra H_2O + *t*-BuCN (C≡N region) . . . S14

S10 FTIR spectra H_2O + *t*-BuCN (C≡N region) + subtraction spectra . . . S15

S11 ORCA input B3LYP/def2-TZVP . . . S17

S12 ORCA input B2PLYP-D3(BJ,abc)/ma-def2-QZVP . . . S17

S13 ORCA input DLPNO-CCSD(T)/ma-def2-QZVP . . . S17

S14 $H_2O\cdots t$-BuCN σ-type xyz coordinates . . . S18
S15 $H_2O\cdots t$-BuCN π-type xyz coordinates . . . S18
S16 t-BuOH$\cdots t$-BuCN σ-type xyz coordinates . . . S19
S17 t-BuOH$\cdots t$-BuCN π-type xyz coordinates . . . S20
S18 $H_2O\cdots t$-BuNC σ-type xyz coordinates . . . S20
S19 $H_2O\cdots t$-BuNC π-type xyz coordinates . . . S21
S20 t-BuOH$\cdots t$-BuNC σ-type xyz coordinates . . . S21
S21 t-BuOH$\cdots t$-BuNC π-type xyz coordinates . . . S22
S22 Gaussian integrals t-BuNC + H_2O + He . . . S28
S23 Gaussian integrals t-BuNC + H_2O + He . . . S28
S24 NCI plots for $H_2O\cdots t$-BuNC . . . S30
S25 NCI plots for t-BuOH$\cdots t$-BuNC . . . S31
S26 NCI plots for $H_2O\cdots t$-BuCN . . . S31
S27 NCI plots for t-BuOH$\cdots t$-BuCN . . . S32
S28 3D plot of computed E_0 differences . . . S33
S29 3D plot of computed E_e differences . . . S34
S30 OHb absolute wavenumbers plot . . . S35
S31 Dipole curves of the N≡C and C≡N stretching modes . . . S35

List of Tables

S1 Filenames of shown spectra . . . S1
S2 Acquisition settings for measurements of the O–H stretching region . . . S2
S3 Acquisition settings for measurement of the C≡N stretching region . . . S2
S4 Details on the chemicals used . . . S3
S5 Experimental OH_b wavenumbers and downshifts . . . S4
S6 Experimental C≡N vibrational wavenumbers and shifts . . . S4
S7 Assigned ro-vibrational water monomer bands . . . S6
S8 Overview of experimental and computed vibrational bands, their intensity and conformer stability . . . S23
S9 Experimental and computed C≡N vibrational wavenumbers, shifts and intensities S23
S10 Overview of computed OH and C≡N wavenumber shifts . . . S24
S11 Computed interaction and dissociation energies . . . S24
S12 Computed electrostatic molecular properties . . . S25

S13 Overview of determined conformational intensity ratios and derived approximated abundance S27
S14 Optimized Gaussian parameters for *t*-BuNC monomer and homodimer bands . S29
S15 Integration of OHb and NC bands S30

1 Experimental Details

1.1 Data availability

The original jet FTIR spectra are made available as dpt-files at the GRO.data repository. The spectra shown in each figure are listed in Table S1.

Table S1: Filenames of all FTIR spectra shown in this work's figures. The filenames are composed of the measurement date (yyyymmdd), the scan batches included in the average (a,b,c,d...), the compounds and carrier gases each followed by their respective partial pressure, the stagnation pressure (p_s) and the number of averaged scans (Av.xxx).

Filename	Figures
20250505n-a-tert-Butylisocyanid-0.4mbar-H2O-0.2mbar-Ne-400mbar-p_s-0,4barpm.800	S1
20250505-abcdefgh-tert-Butylisocyanid-0.2mbar-H2O-0.4mbar-Ne-400mbar-p_s-0,4barpm-Av.720	S1, S23
20250506-abcde-tert-Butylisocyanid-0.2mbar-H2O-0.4mbar-He-400mbar-p_s-0,4barpm-Av.800	3, S1, S22, S30
20250507-abcde-tert-Butylisocyanid-0.4mbar-tBuOH-0.2mbar-Ne-400mbar-p_s-0,4barpm-Av.1000	S2
20250507n-a-tert-Butylisocyanid-0.2mbar-tBuOH-0.4mbar-Ne-400mbar-p_s-0,4barpm.900	S2
20250508-abcdef-tert-Butylisocyanid-0.2mbar-tBuOH-0.4mbar-He-400mbar-p_s-0,4barpm-Av.750	3, S2, S30
20250404-abcd-Pivalonitrile-0,4mbar-H2O-0,2mbar-Ne-400mbar-p_s-0,4barpm-Av.800	S3
20250407-abcdef-Pivalonitrile-0,2mbar-H2O-0,4mbar-Ne-400mbar-p_s-0,4barpm-Av.800	S3
20250411-abc-Pivalonitrile-0,2mbar-H2O-0,4mbar-He-400mbar-p_s-0,4barpm-Av.800	3, S3, S30
20250422-abcde-Pivalonitrile-0.4mbar-tBuOH-0.2mbar-Ne-400mbar-p_s-0,4barpm-Av.750	S4
20250417-abc-Pivalonitrile-0.2mbar-tBuOH-0.4mbar-Ne-400mbar-p_s-0,4barpm-Av.800	S4
20250422n-a-Pivalonitrile-0.2mbar-tBuOH-0.4mbar-He-400mbar-p_s-0,4barpm.800	3, S4, S30
20250925-abcde-tert-Butylisocyanide-0.400mbar-H2O-0.200mbar-Ne-400mbar-p_s-0,4barpm-Av.850	S5, S6
20250926-abcd-tert-Butylisocyanide-0.200mbar-H2O-0.400mbar-Ne-400mbar-p_s-0,4barpm-Av.800	4, S5
20250930-abcd-tert-Butylisocyanide-0.400mbar-Ne-400mbar-p_s-0,4barpm-Av.450	S5, S6
20250728a-abc-Pivalonitrile-0.400mbar-Ne-400mbar-p_s-0,4barpm-Av.350	S7, S8
20250728-abc-Pivalonitrile-0.800mbar-Ne-400mbar-p_s-0,4barpm-Av.350	S7, S8
20250923-abcd-Pivalonitrile-0.400mbar-H2O-0.800mbar-Ne-400mbar-p_s-0,4barpm-Av.850	4, S9, S10
20250924-abcde-Pivalonitrile-0.800mbar-H2O-0.400mbar-Ne-400mbar-p_s-0,4barpm-Av.850	S9, S10

1.2 Instrumentation

The experiments within this work were conducted exclusively using *gratin*-jet spectroscopy, an acronym for gas-recycling atom economic infrared jet spectroscopy. This experimental setup has been described previously.[1–3] In brief, the setup combines Fourier-transform infrared spectroscopy (FTIR) with supersonic expansions to acquire jet-cooled IR spectra, while enabling gas-recycling through compressing the gas back to the reservoir after every pulse.

For each experiment, a gas mixture consisting of 0.2–0.8 mbar analyte(s) and 400 mbar carrier gas was introduced into a 0.2 m^3 reservoir. This gas mixture was then expanded through a 0.2 mm $\times$ 700 mm slit nozzle into a pre-evacuated 4 m^3 buffer volume. The

molecules and molecular clusters were probed during expansion by a mildly focused IR beam from a Bruker Vertex 70v FTIR spectrometer, with the signal detected by a liquid-N_2-cooled InSb/HgCdTe sandwich detector. Signal processing was handled by Opus (v. 7.8), Bruker's integrated software. After each pulse, the gas mixture was recompressed for reuse. Acquisition settings are summarized in Table S2 and Table S3.

Table S2: Acquisition settings used for most spectra recorded in this work. These settings lead to an optimized signal-to-noise ratio above 3000 cm^{-1}.
Abbreviations: "LN" – Liquid Nitrogen, "SW" – Sandwich Detector, "NBM" – Norton-Beer Medium, "ML" – Mertz Left, "ds" – Double Sided.

Setting	Specification	Setting	Specification
Detector	LN-InSb SW	Scanner Velocity	140 kHz, ds
Beamsplitter	CaF_2	Aperture Size	5.0 mm
Light Source	Tungsten 20 W	Aperture Filter	F20
Apodization Function	NBM	Average Nozzle Distance	10 mm
Phase Correction Mode	ML	Delay/Ontime	146/133 ms
Zero Filling	$\times 4$	Optical Resolution	2 cm^{-1}

Table S3: Acquisition settings used for globar-measurements recorded in this work. Settings that are not specified are identical to those given in Table S2.

Setting	Specification
Light Source	Globar (SiC)
Aperture Size	6.0 mm

1.3 Investigated Compounds

The chemicals used in this work are listed in Table S4. All analytes were purified by a single *freeze-pump-thaw-cycle*.[1] To prevent photoinduced polymerization, *tert*-butyl isocyanide was protected from prolonged exposure to light.

Table S4: List of chemicals used, including their formulae, CAS numbers, suppliers, and purities.

Compound	Formula	CAS No.	Supplier	Purity
Helium	He	7440-59-7	Nippon Gases	$\geq 99.999\,\%$
Neon	Ne	7440-01-9	Linde Gas	$\geq 99.999\,\%$
Water	H_2O	7732-18-5	——	Demineralised
tert-Butyl alcohol	*t*-BuOH	75-65-0	TCI Chemicals	$\geq 99.8\,\%$
Pivalonitrile	*t*-BuCN	630-18-2	Angene	$\geq 99.0\,\%$
tert-Butyl isocyanide	*t*-BuNC	7188-38-7	Angene	$\geq 98.0\,\%$

1.4 FTIR Spectra

The spectra in this work are plotted using the `Matplotlib`[4] (v. 3.8.4) library in Python[5] (v. 3.11.9). Data processing is achieved with `NumPy`[6] (v. 1.26.4) and `BrukerOpus`.[7]

The cluster composition of an observed signal is determined by analyzing the scaling behavior upon varying analyte partial pressures. This assignment is achieved through comparison of signal intensities at donor/acceptor partial pressure ratios of 0.2 mbar/0.4 mbar and 0.4 mbar/0.2 mbar. Since the signal strength is proportional to the abundance of the probed species as stated by the Beer-Lambert law,[8] the signal strength of a 1:1 dimer will stay approximately constant between the measurements as the product of partial pressures stays the same. In contrast, the signal strength of a 2:1 (1:2) dimer will roughly double (halve) between the measurements, when switching from a 1:2 partial pressure ratio to a 2:1 ratio. Note that exceptions may occur due to spectral overlap or non-linear scaling behavior. Because 1:1 dimers and 2:2 tetramers are not distinguishable by this approach, a change of expansion conditions is needed for final assignment. While colder expansions in Ne favor larger assemblies, warmer He expansions counteract larger cluster formation because of less efficient collisions, affecting higher-order clusters more than smaller assemblies.

Table S5: Overview of experimentally determined OH_b wavenumbers $\tilde{\nu}_{exp}$ and downshifts $-\Delta\tilde{\nu} = \tilde{\nu}_{mono} - \tilde{\nu}_{dim}$ of assigned dimer bands from the monomer band.

	Dimer	Type	$\tilde{\nu}_{exp}$ / cm^{-1}	$-\Delta\tilde{\nu}$ / cm^{-1}	Spectrum
**A**	$H_2O\cdots t$-BuNC	σ	3565(1)	92(1)	Figure S1
		π	—	—	
**B**	t-BuOH$\cdots t$-BuNC	σ	3534(1)	108(1)	Figure S2
		π	3596(1)	46(1)	
**C**	$H_2O\cdots t$-BuCN	σ	3595(1)	62(1)	Figure S3
		π	3613(1)	44(1)	
**D**	t-BuOH$\cdots t$-BuCN	σ	—	—	Figure S4
		π	3590(1)	52(1)	

Table S6: Overview of experimentally determined C≡N vibrational wavenumbers $\tilde{\nu}_{exp}$ and shifts $\Delta\tilde{\nu} = \tilde{\nu}_{dim} - \tilde{\nu}_{mono}$ of assigned dimer bands from the monomer band.

Species	$\tilde{\nu}_{exp}$ / cm^{-1}	$\Delta\tilde{\nu}$ / cm^{-1}	Spectrum
t-BuNC	2141(1)	—	Figure S5
t-BuNC$\cdots t$-BuNC	2148(1)	+7(2)	
$H_2O\cdots t$-BuNC (σ)	2160(1)	+19(2)	
t-BuCN	2246(1)	—	Figure S8
t-BuCN$\cdots t$-BuCN	2244(1)	−2(2)	

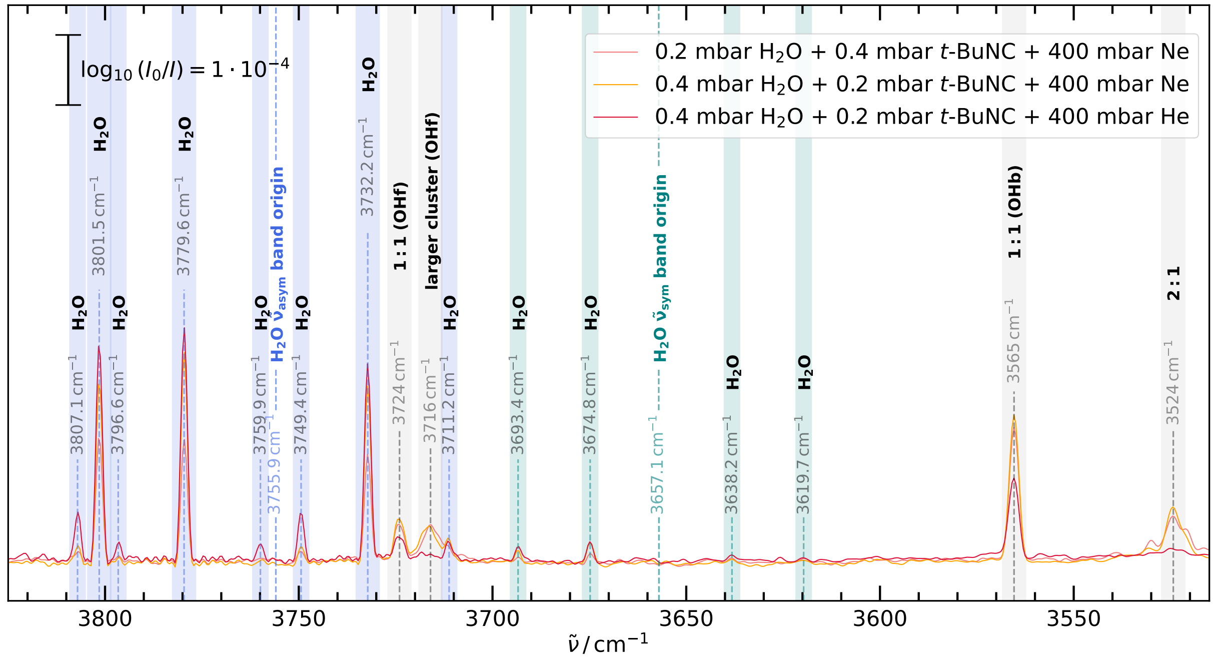

Figure S1: FTIR spectra of water and *tert*-butyl isocyanide in the 3820–3520 cm^{-1} region with varying experimental conditions to assess cluster stoichiometry (donor:acceptor). Acquisition settings were used according to Table S2. The assignments derived through scaling behavior along with the determined vibrational wavenumber are also given. Ro-vibrational water monomer transitions of the asymmetric (blue) and symmetric (teal) stretching fundamental show a more complex scaling behavior because they are sensitive to rotational temperature and spectral resolution. They are identified by comparison to literature values (see Table S7).

Table S7: Assigned ro-vibrational water monomer bands $\tilde{\nu}_{exp}$ as given in Figure S1 in comparison with literature values[9] $\tilde{\nu}_{lit}$. Due to laser calibration, partial overlaps and limited interferometric resolution assigned water lines have an RMSD to literature values of 0.13 cm^{-1}. Experimental uncertainties are given in parentheses. Transitions are denoted using following nomenclature: rotational term quantum numbers as $J_{K_aK_c}$, symmetric and asymmetric vibrational fundamental transitions ($v' \leftarrow v = 1 \leftarrow 0$) as $\tilde{\nu}_{sym}$ and $\tilde{\nu}_{asym}$.

Fundamental	$J'_{K_a'K_c'} \leftarrow J_{K_aK_c}$	$\tilde{\nu}_{exp}$ / cm^{-1}	$\tilde{\nu}_{lit}$ / cm^{-1}
$\tilde{\nu}_{asym}$	$2_{11} \leftarrow 1_{10}$	3807.1(1)	3807.0149(5)
$\tilde{\nu}_{asym}$	$2_{02} \leftarrow 1_{01}$	3801.5(1)	3801.4196(5)
$\tilde{\nu}_{asym}$	$2_{12} \leftarrow 1_{11}$	3796.6(1)	3796.4400(5)
$\tilde{\nu}_{asym}$	$1_{01} \leftarrow 0_{00}$	3779.6(1)	3779.4938(5)
$\tilde{\nu}_{asym}$	$1_{10} \leftarrow 1_{11}$	3759.9(1)	3760.1251(5)
$\tilde{\nu}_{asym}$	$1_{11} \leftarrow 1_{10}$	3749.4(1)	3749.3302(5)
$\tilde{\nu}_{asym}$	$0_{00} \leftarrow 1_{01}$	3732.2(1)	3732.1354(5)
$\tilde{\nu}_{asym}$	$2_{12} \leftarrow 1_{01}$	3711.2(1)	3711.1030(5)
$\tilde{\nu}_{sym}$	$1_{11} \leftarrow 0_{00}$	3693.4(1)	3693.2940(5)
$\tilde{\nu}_{sym}$	$1_{10} \leftarrow 1_{01}$	3674.8(1)	3674.6971(5)
$\tilde{\nu}_{sym}$	$1_{11} \leftarrow 1_{01}$	3638.2(1)	3638.0822(5)
$\tilde{\nu}_{sym}$	$0_{00} \leftarrow 1_{11}$	3619.7(1)	3619.9163(5)

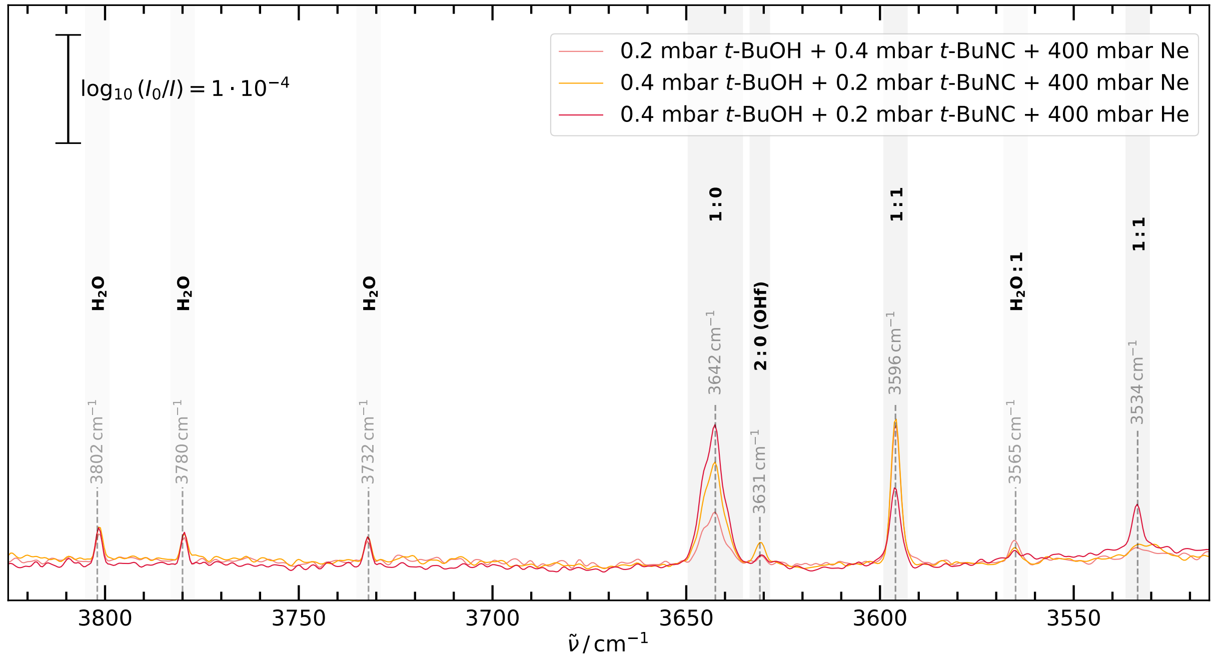

Figure S2: FTIR spectra of *tert*-butyl alcohol and *tert*-butyl isocyanide in the 3820–3520 cm^{-1} region with varying experimental conditions to assess cluster stoichiometry (donor:acceptor). Acquisition settings were used according to Table S2. The assignments derived through scaling behavior along with the determined vibrational wavenumber are also given.

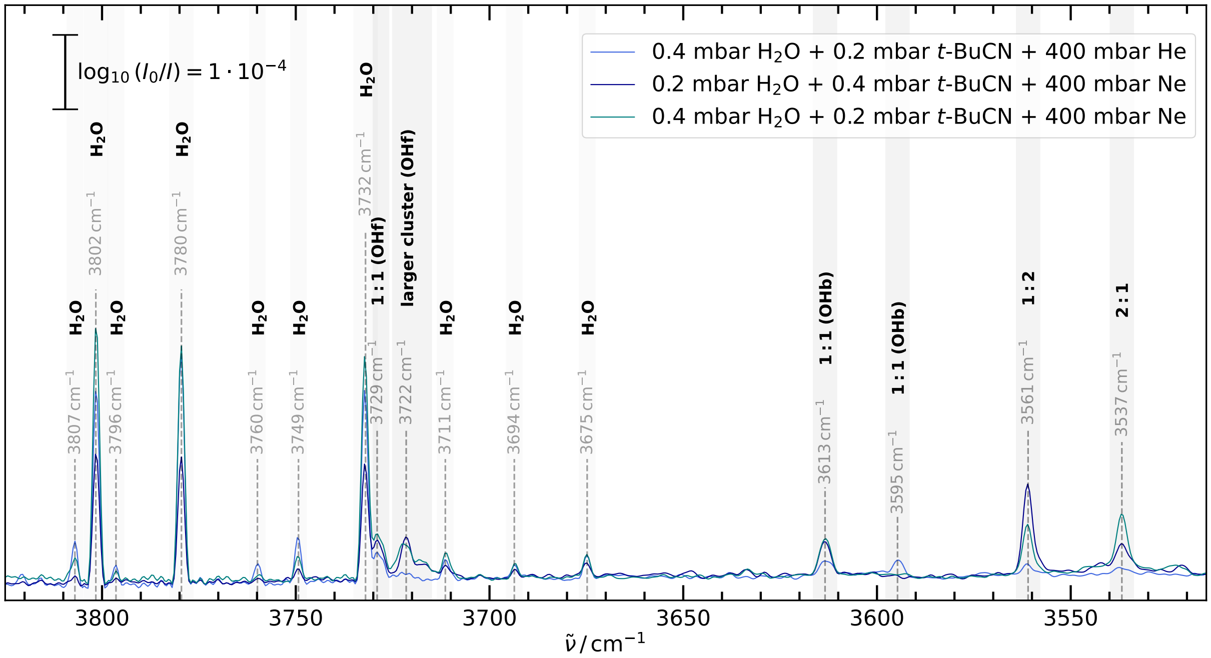

Figure S3: FTIR spectra of water and pivalonitrile in the 3820–3520 cm^{-1} region with varying experimental conditions to assess cluster stoichiometry (donor:acceptor). Acquisition settings were used according to Table S2. The assignments derived through scaling behavior along with the determined vibrational wavenumber are also given.

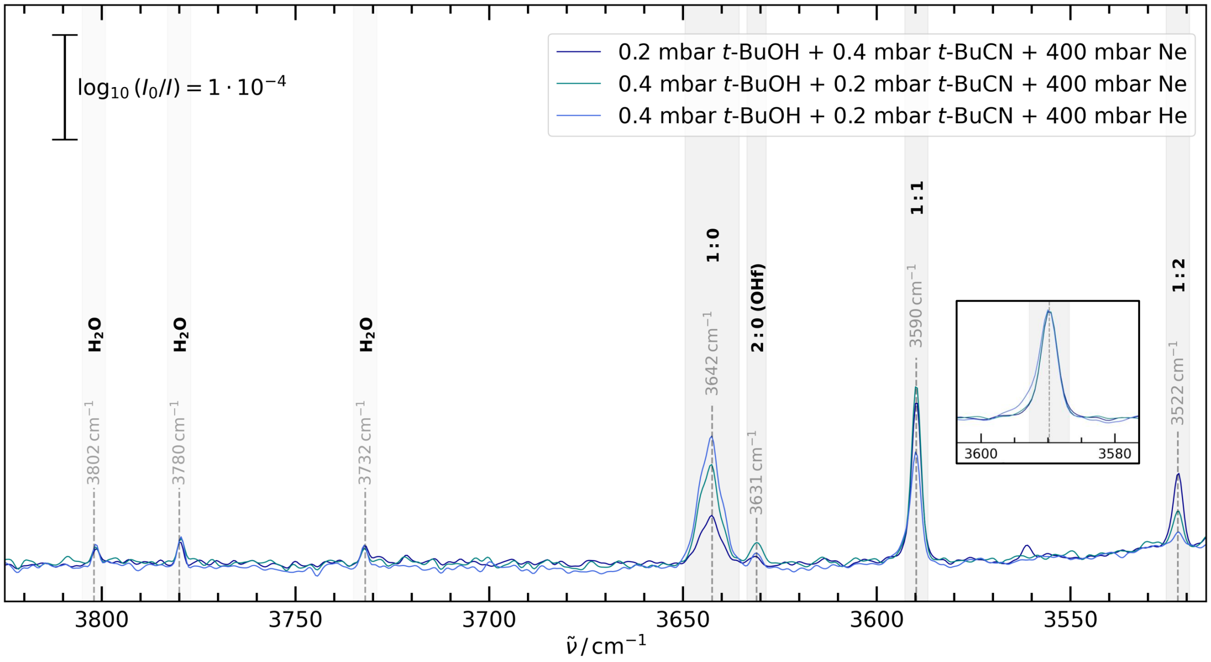

Figure S4: FTIR spectra of *tert*-butyl alcohol and pivalonitrile in the 3820–3520 cm^{-1} region with varying experimental conditions to assess cluster stoichiometry (donor:acceptor). Acquisition settings were used according to Table S2. The assignments derived through scaling behavior along with the determined vibrational wavenumber are also given. When scaled to uniform peak height, a shoulder of the 1:1 band in He is indicated at higher frequencies. This is visualized in the insert. This could be due to a small fraction of a metastable complex isomer, but also due to thermal excitation of the dominant complex isomer. If the shoulder of the 1:1 band, indicated above, belonged to the σ-type complex, its share of $<$1 %, estimated in the main text using Equation S2, would be higher. However, through a comparison with other spectra and theoretical performance, the σ-type is not expected to cause a smaller wavenumber downshift of the OHb than the π-type. Therefore, the maximum share of the σ-type is based on noise level rather than on the shoulder intensity. One should note, however, that the asymmetric lineshape only appears with He as the carrier gas and only for this particular band.

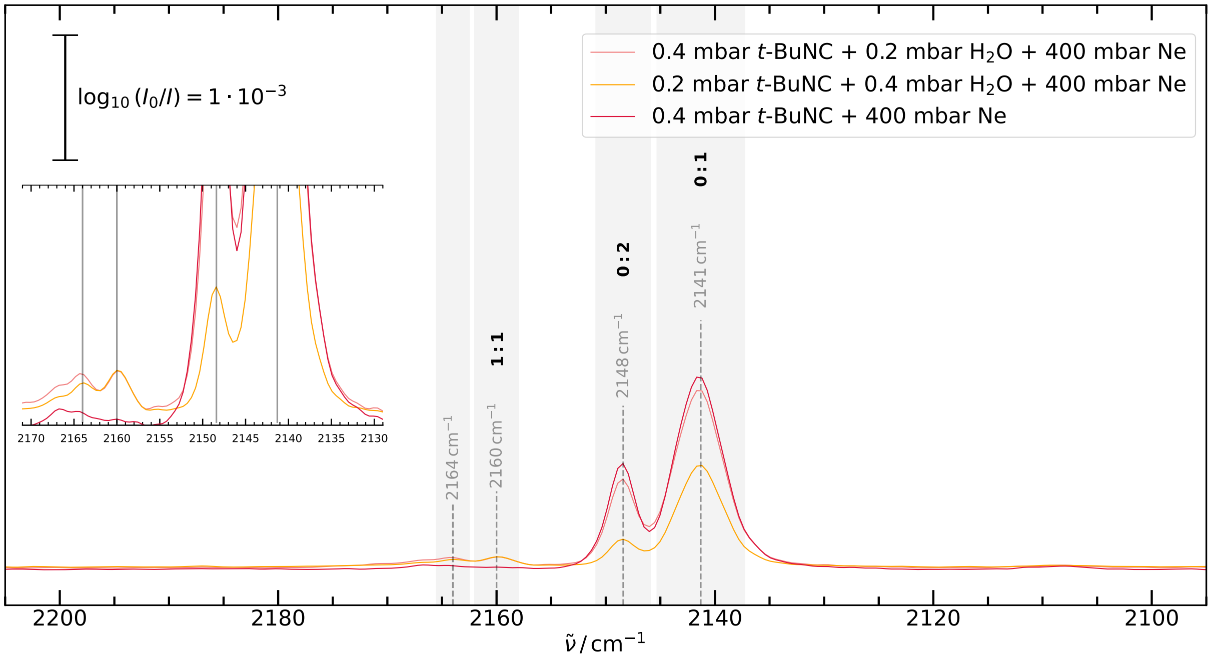

Figure S5: FTIR spectra of *tert*-butyl isocyanide and water in the 2200–2100 cm^{-1} region with varying experimental conditions to assess cluster stoichiometry (donor:acceptor). Acquisition settings were used according to Table S2. The assignments derived through scaling behavior along with the determined vibrational wavenumber are also given.

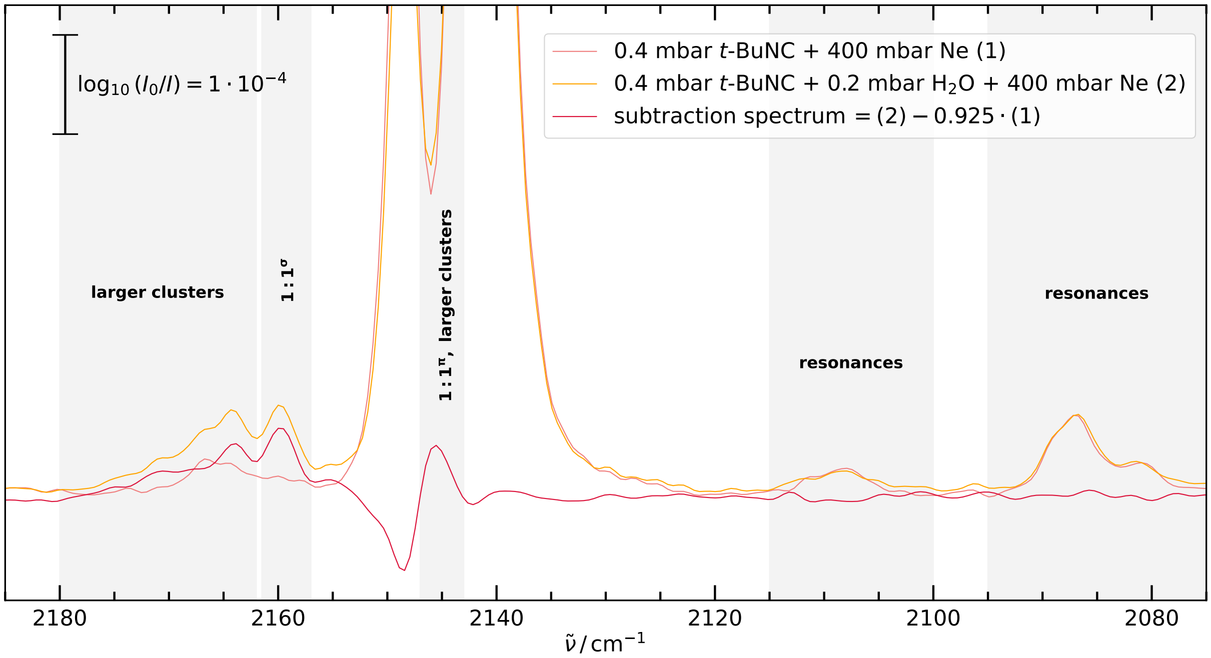

Figure S6: FTIR spectra of *tert*-butyl isocyanide with and without water in the 2180–2080 cm^{-1} region. Acquisition settings were used according to Table S3. Additionally, a monomer subtraction spectrum is shown in order to highlight the minimum plausible amount of heterocluster features and weak monomer resonances. Signals which cancel are likely due to isocyanide monomer, signals which remain positive are due to hydrate clusters and signals which become negative are likely due to isocyanide homoclusters.

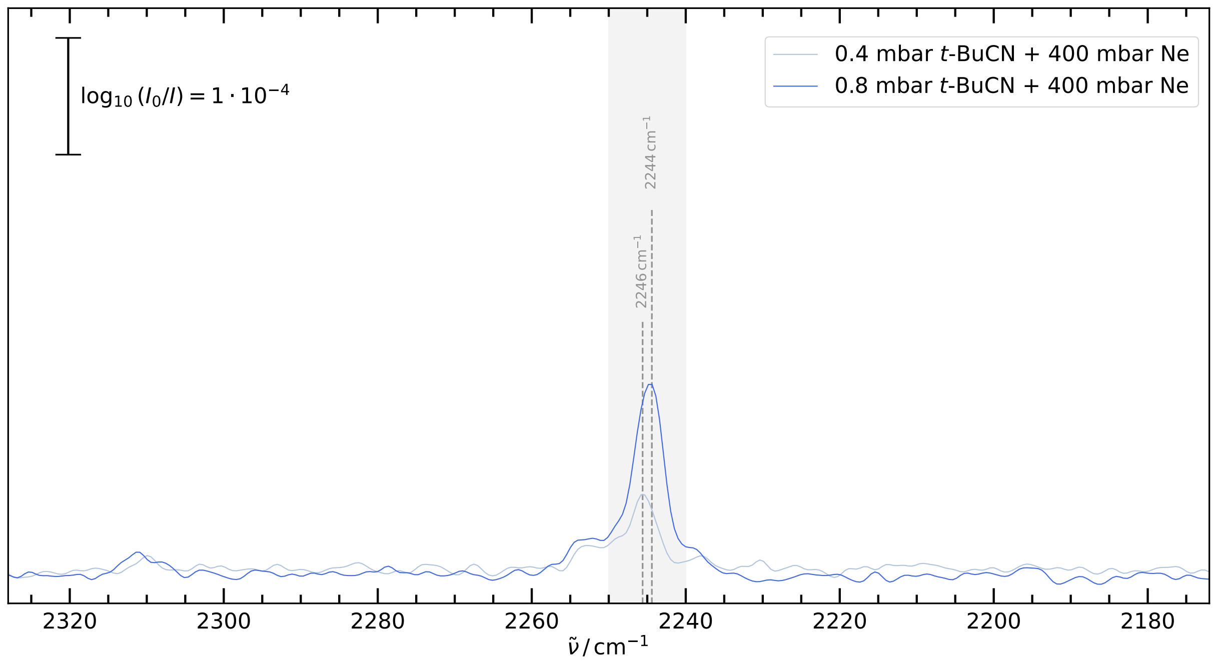

Figure S7: FTIR spectra of pivalonitrile in the 2330–2170 cm^{-1} region with varying analyte concentration. Acquisition settings were used according to Table S3. The respective determined absorption maxima are marked with their vibrational wavenumber.

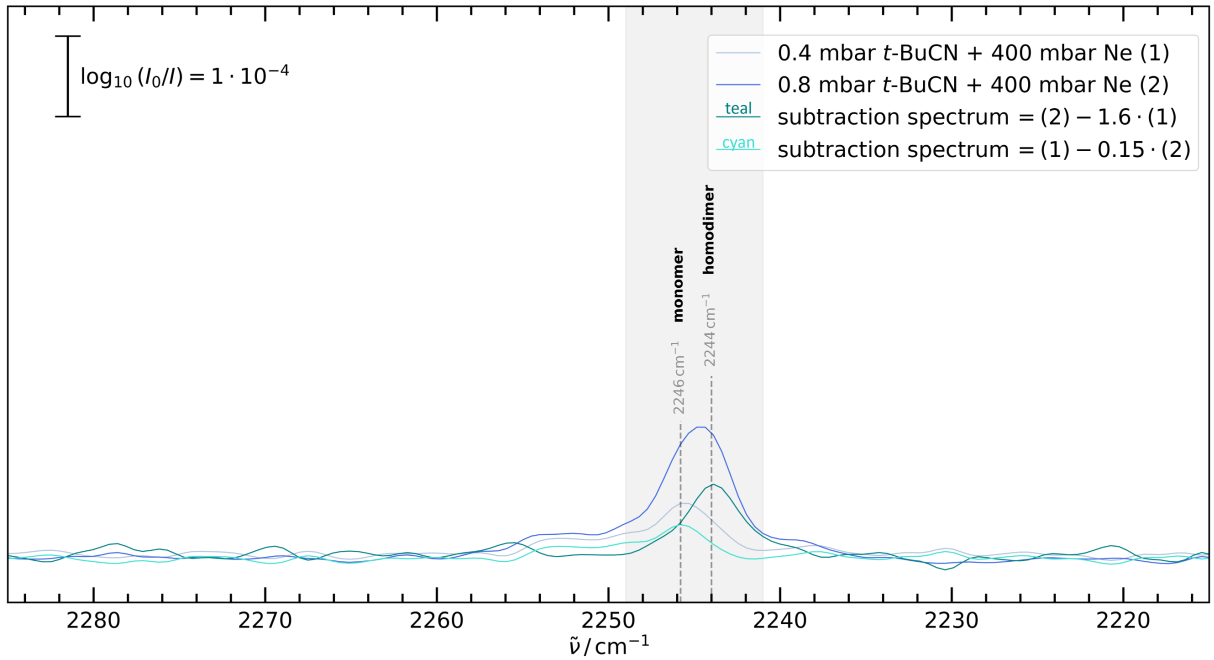

Figure S8: FTIR spectra of pivalonitrile in the 2280–2220 cm^{-1} region with varying analyte concentration. Acquisition settings were used according to Table S3. Additionally, subtraction spectra are shown to separate the peak contributions into monomer (cyan) and dimer (teal) contributions.

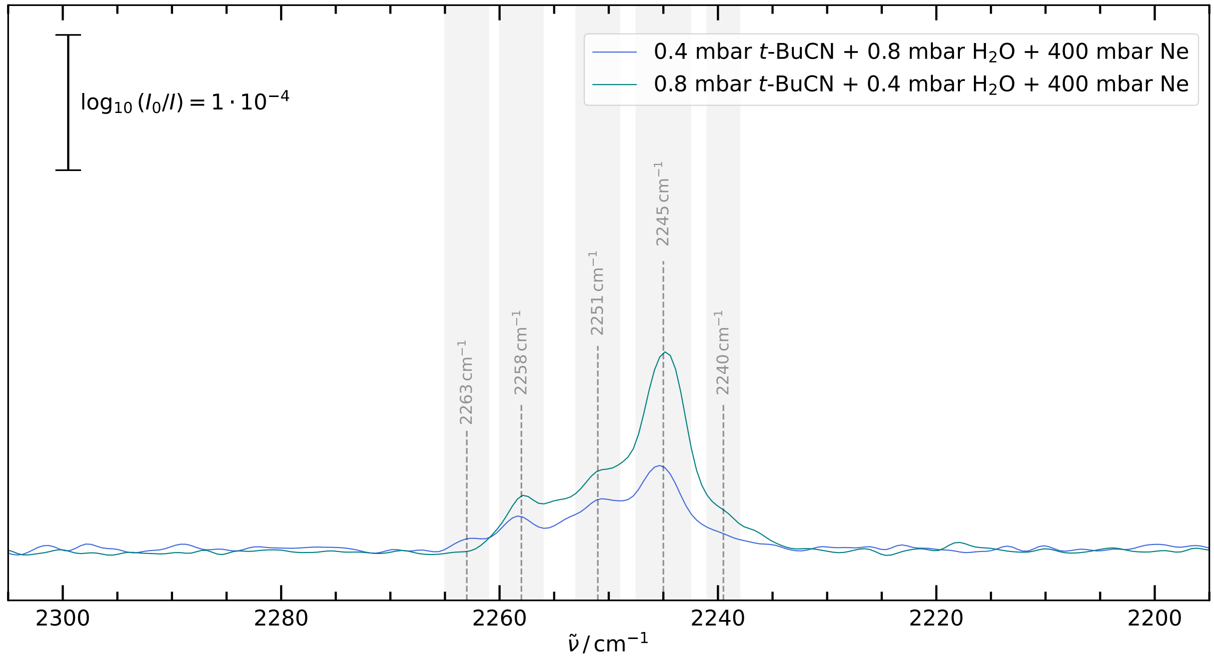

Figure S9: FTIR spectra of pivalonitrile and water in the 2300–2200 cm^{-1} region with varying analyte concentration. Acquisition settings were used according to Table S3. The respective determined absorption maxima are marked with their vibrational wavenumber.

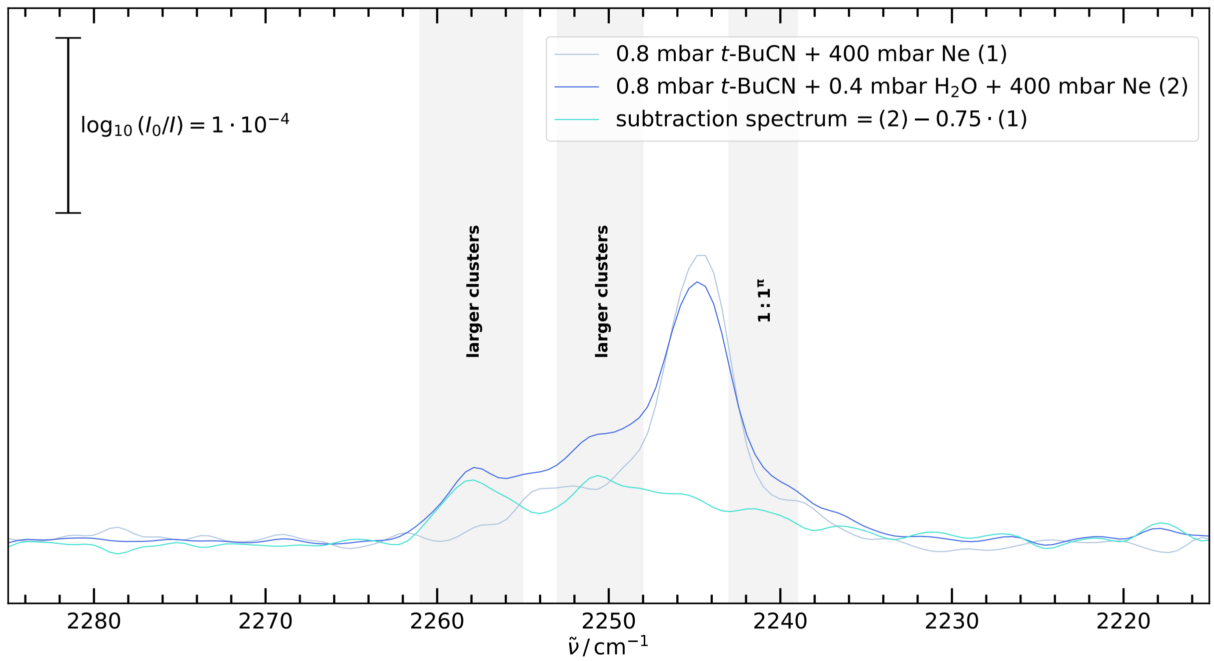

Figure S10: FTIR spectra of pivalonitrile with and without water in the 2280–2220 cm^{-1} region. Acquisition settings were used according to Table S3. Additionally, a mixed spectrum with conservative nitrile-only subtraction is shown in order to highlight the maximum plausible extent of heterocluster features.

2 Computational Details

2.1 Methods

The computations in this work were exclusively performed using ORCA[10] (v. 5.0.4[11]).

CREST[12,13] (v. 3.0.2) was used in connection with the semi-empirical GFN2-xTB method[14,15] (v. 6.7.1) for initial conformer sampling. For re-optimization, the B3LYP hybrid functional was employed, using the canonical 20 % HF exchange, 72 % Becke 1988 exchange and 81 % Lee-Yang-Parr correlation.[16–19] Grimme's D3 dispersion correction[20] with Becke-Johnson damping[21–24] (D3(BJ)) was applied throughout, including a three-body (abc) term. As a basis set, def2-TZVP,[25] a triple-ζ Karlsruhe basis set with valence polarization functions, was used. From these less expensive calculations, harmonic wavenumbers were extracted, which after multiplying by the scaling factor in this work (0.97) yielded predictions for the experimentally determined wavenumbers.

For more accurate structure optimizations, the double-hybrid B2PLYP functional[26,27] with 53 % HF exchange and 27 % MP2 correlation, was utilized. In these calculations, the D3(BJ,abc) dispersion correction was applied as well. For this method, the minimally augmented ma-def2-QZVP basis set[25,28] with quadruple-ζ quality and additional diffuse functions was used. Additionally, ORCA's auxiliary basis sets – def2/J for exchange and def2-QZVPP/C for correlation – were used to accelerate the computations via the resolution-of-the-identity (RI) approximation.

For even more accurate single point computations, DLPNO-CCSD(T) was the method of choice, which uses the coupled cluster method[29,30] including single (S) and double (D) excitations, while treating triple excitations ((T)) perturbatively. To scale down the computational cost, the domain-based local pair natural orbital (DLPNO) approximation[31] was used as well as ORCA's auxiliary basis sets same as for B2PLYP calculations. Note that geometry optimizations or harmonic wavenumber computations were not performed with this method. Instead, the B2PLYP-D3(BJ,abc)/ma-def2-QZVP geometries were used for DLPNO-CCSD(T) single point calculations. B2PLYP-D3(BJ,abc)/ma-def2-QZVP wavenumber computations were used for harmonic zero-point correction of the DLPNO-CCSD(T) energies. This approach is termed in this work as the DLPNO-CCSD(T)//B2PLYP-D3(BJ,abc)/ma-def2-QZVP composite method.

The ORCA input files for the methods presented are given in subsection 2.2.

2.2 Input Files

```
! B3LYP D3BJ ABC def2-TZVP DefGrid3 VeryTightSCF VeryTightOpt Freq Mass2016
%geom
   EnforceStrictConvergence True
   MaxIter 300
end
%pal nprocs 12 end
%Maxcore 4000

    *xyzfile 0 1 tBuCN_H2O_D_lin.xyz
```

Figure S11: ORCA input for a geometry optimization followed by a harmonic frequency calculation at the B3LYP-D3(BJ,abc)/def2-TZVP level of computation.

```
! B2PLYP D3BJ ABC ma-def2-QZVP def2/J def2-QZVPP/C DefGrid3 TightSCF TightOpt NumFreq Mass2016
%geom
    EnforceStrictConvergence True
    MaxIter 300
end
%scf
    MaxIter 1500
end
%pal nprocs 12 end
%Maxcore 4000

*xyzfile 0 1 tBuCN_H2O_D_lin.xyz
```

Figure S12: ORCA input for a geometry optimization followed by a harmonic numerical frequency calculation at the B2PLYP-D3(BJ,abc)/ma-def2-QZVP level of computation.

```
! DLPNO-CCSD(T) ma-def2-QZVP def2/J def2-QZVPP/C TightSCF TightPNO LED
%mdci
    LocMaxIterLed 3000
end
%pal nprocs 12 end
%Maxcore 4000

*xyzfile 0 1 tBuCN_H2O_D_lin.xyz
```

Figure S13: ORCA input for a single point calculation at the DLPNO-CCSD(T)/ma-def2-QZVP level of computation.

2.3 Structure Output Data

```
18
symmetry c1
N        1.311433000     -0.336452000      0.080029000
C        0.166878000     -0.181004000      0.053520000
C       -1.287249000      0.022271000      0.017080000
C       -1.767297000      0.372882000      1.432787000
H       -1.292257000      1.282409000      1.791929000
H       -2.844172000      0.528629000      1.414748000
H       -1.545772000     -0.430737000      2.130757000
C       -1.592381000      1.174006000     -0.951596000
H       -1.115465000      2.094970000     -0.625710000
H       -1.244582000      0.942594000     -1.955282000
H       -2.668233000      1.334044000     -0.985908000
C       -1.950874000     -1.274626000     -0.467720000
H       -1.731483000     -2.100730000      0.204201000
H       -3.029214000     -1.131913000     -0.499917000
H       -1.607408000     -1.540007000     -1.464508000
H        3.320755000     -0.041504000     -0.080483000
O        4.258558000      0.167000000     -0.179589000
H        4.714602000     -0.636162000      0.073335000
```

Figure S14: Cartesian coordinates (XMOL format) of σ-type $H_2O\cdots t$-BuCN computed at the B2PLYP-D3(BJ,abc)/ma-def2-QZVP level of computation.

```
18
symmetry c1
N        0.958954000      1.814354000     -0.041422000
C        0.095770000      1.043752000     -0.013353000
C       -0.962468000      0.025911000      0.021366000
C       -0.837471000     -0.839160000     -1.241958000
H       -0.971535000     -0.239782000     -2.139526000
H        0.137278000     -1.317811000     -1.282936000
H       -1.608044000     -1.607642000     -1.221412000
C       -0.756084000     -0.837076000      1.275430000
H       -0.828910000     -0.235686000      2.178692000
H       -1.527970000     -1.603848000      1.307562000
H        0.218234000     -1.317844000      1.252723000
C       -2.326406000      0.727914000      0.064701000
H       -3.111991000     -0.024793000      0.089337000
H       -2.418181000      1.351574000      0.950781000
H       -2.473972000      1.351903000     -0.813565000
H        2.418840000      0.112221000     -0.091066000
O        2.561365000     -0.840450000     -0.096843000
H        3.512041000     -0.955457000     -0.129372000
```

Figure S15: Cartesian coordinates (XMOL format) of π-type $H_2O\cdots t$-BuCN computed at the B2PLYP-D3(BJ,abc)/ma-def2-QZVP level of computation.

```
30
symmetry c1
C        1.377349000     -0.233156000      0.125874000
N        0.242488000     -0.443895000      0.181658000
C        2.819655000      0.033665000      0.052112000
C        3.303888000      0.492179000      1.435075000
H        3.126071000     -0.274424000      2.185169000
H        4.372903000      0.690580000      1.389753000
H        2.796309000      1.402371000      1.744878000
C        3.533855000     -1.260658000     -0.363059000
H        3.360594000     -2.052136000      0.361806000
H        3.188519000     -1.602350000     -1.335628000
H        4.604159000     -1.073024000     -0.421832000
C        3.058860000      1.134787000     -0.991003000
H        4.125821000      1.339330000     -1.054026000
H        2.707222000      0.825991000     -1.972297000
H        2.546589000      2.052992000     -0.714313000
O       -2.687633000     -1.177155000     -0.066287000
C       -3.416820000     -0.042176000      0.406932000
C       -4.884524000     -0.413144000      0.260531000
C       -3.082000000      1.179729000     -0.447531000
C       -3.070632000      0.219256000      1.872006000
H       -1.744610000     -0.985258000      0.013378000
H       -5.114162000     -0.626038000     -0.781387000
H       -5.105242000     -1.302060000      0.847613000
H       -5.524843000      0.398694000      0.601005000
H       -2.022425000      1.418577000     -0.367556000
H       -3.309773000      0.976966000     -1.491982000
H       -3.653816000      2.050579000     -0.128569000
H       -3.638221000      1.062001000      2.265343000
H       -3.295132000     -0.661478000      2.470094000
H       -2.009869000      0.443114000      1.975960000
```

Figure S16: Cartesian coordinates (XMOL format) of σ-type t-BuOH$\cdots t$-BuCN computed at the B2PLYP-D3(BJ,abc)/ma-def2-QZVP level of computation.

```
30
symmetry c1
C       -1.734057000      1.102109000      0.060169000
N       -1.124788000      2.058913000     -0.170891000
C       -2.466403000     -0.135240000      0.360074000
C       -2.030688000     -0.627413000      1.748578000
H       -2.560829000     -1.550078000      1.977937000
H       -0.961355000     -0.820873000      1.767405000
H       -2.268938000      0.106763000      2.514823000
C       -3.971585000      0.163110000      0.337562000
H       -4.233410000      0.911871000      1.081373000
H       -4.285296000      0.522762000     -0.639593000
H       -4.517871000     -0.751233000      0.560832000
C       -2.104191000     -1.179689000     -0.706124000
H       -2.638835000     -2.103388000     -0.492222000
H       -2.386730000     -0.838821000     -1.699620000
H       -1.036080000     -1.378757000     -0.692312000
O        1.148824000     -0.047547000      0.365440000
C        2.290055000     -0.107288000     -0.505178000
C        2.917738000     -1.467990000     -0.250475000
C        3.259263000      1.015544000     -0.147911000
C        1.829385000      0.018770000     -1.954911000
H        0.711375000      0.800478000      0.230551000
H        3.801129000     -1.605810000     -0.870637000
H        3.207844000     -1.558123000      0.794039000
H        2.206757000     -2.259412000     -0.478943000
H        2.787822000      1.986752000     -0.295167000
H        3.558905000      0.933901000      0.894561000
H        4.151086000      0.975242000     -0.771553000
H        1.131168000     -0.779423000     -2.199072000
H        1.328400000      0.972868000     -2.114430000
H        2.674434000     -0.040121000     -2.639223000
```

Figure S17: Cartesian coordinates (XMOL format) of π-type t-BuOH$\cdots t$-BuCN computed at the B2PLYP-D3(BJ,abc)/ma-def2-QZVP level of computation.

```
18
symmetry c1
C        1.477915000      0.418517000      0.009221000
N        0.355594000      0.099860000      0.010496000
C       -1.027003000     -0.306810000      0.008949000
C       -1.219046000     -1.310627000     -1.127645000
H       -2.256725000     -1.635435000     -1.150888000
H       -0.973500000     -0.857756000     -2.084764000
H       -0.583997000     -2.180566000     -0.981830000
C       -1.328977000     -0.948468000      1.362984000
H       -0.695328000     -1.816481000      1.525526000
H       -1.161052000     -0.239303000      2.169448000
H       -2.368997000     -1.265557000      1.387972000
C       -1.883631000      0.940008000     -0.209642000
H       -1.644036000      1.407908000     -1.161052000
H       -2.934490000      0.659902000     -0.213311000
H       -1.718397000      1.662918000      0.585113000
H        3.597816000      0.688001000     -0.106554000
O        4.560570000      0.746155000     -0.179747000
H        4.775281000      1.628209000      0.125342000
```

Figure S18: Cartesian coordinates (XMOL format) of σ-type $H_2O\cdots t$-BuNC computed at the B2PLYP-D3(BJ,abc)/ma-def2-QZVP level of computation.

```
18
symmetry c1
C        0.818449000     -1.913942000      0.016301000
N        0.028203000     -1.052998000      0.012774000
C       -0.902774000      0.049417000      0.007282000
C       -0.653949000      0.862643000     -1.263310000
H        0.365928000      1.236912000     -1.277748000
H       -0.819548000      0.251295000     -2.147188000
H       -1.341572000      1.705202000     -1.289308000
C       -2.316498000     -0.530140000      0.025783000
H       -2.488058000     -1.152951000     -0.848633000
H       -2.473905000     -1.131314000      0.917828000
H       -3.038627000      0.283109000      0.021572000
C       -0.633225000      0.892791000      1.253748000
H       -1.320851000      1.735565000      1.271165000
H       -0.783578000      0.302673000      2.154617000
H        0.386498000      1.267612000      1.242192000
H        2.481330000     -0.204033000     -0.018000000
O        2.671909000      0.739213000     -0.032198000
H        3.628217000      0.801316000     -0.048859000
```

Figure S19: Cartesian coordinates (XMOL format) of π-type $H_2O\cdots t$-BuNC computed at the B2PLYP-D3(BJ,abc)/ma-def2-QZVP level of computation.

```
30
symmetry c1
C       -1.490166000     -0.126809000     -4.080064000
N       -0.897781000      0.250114000     -3.148064000
C       -0.173227000      0.713253000     -1.991592000
C       -0.759203000      2.064200000     -1.581669000
C       -0.351484000     -0.322146000     -0.881456000
C        1.298101000      0.849627000     -2.382752000
H       -1.813267000      1.965674000     -1.335389000
H       -0.229778000      2.437457000     -0.708073000
H       -0.656624000      2.786686000     -2.387229000
H       -1.402589000     -0.436800000     -0.629557000
H        0.038925000     -1.287942000     -1.191860000
H        0.186795000      0.003228000      0.005771000
H        1.868679000      1.199085000     -1.525379000
H        1.700266000     -0.108414000     -2.702091000
H        1.414523000      1.563845000     -3.193829000
O       -3.195973000     -1.064497000     -6.535406000
C       -2.359972000     -1.886735000     -7.352223000
C       -3.234055000     -2.325412000     -8.517181000
C       -1.884023000     -3.098425000     -6.551552000
C       -1.165634000     -1.074906000     -7.852428000
H       -2.681757000     -0.766654000     -5.772270000
H       -4.096621000     -2.877412000     -8.149947000
H       -3.592462000     -1.455681000     -9.063742000
H       -2.676347000     -2.962774000     -9.201180000
H       -1.285966000     -2.778642000     -5.699381000
H       -2.739891000     -3.657290000     -6.178764000
H       -1.275483000     -3.761005000     -7.166195000
H       -0.533300000     -1.670822000     -8.509867000
H       -1.513084000     -0.201669000     -8.400609000
H       -0.560446000     -0.734254000     -7.013461000
```

Figure S20: Cartesian coordinates (XMOL format) of σ-type t-BuOH$\cdots t$-BuNC computed at the B2PLYP-D3(BJ,abc)/ma-def2-QZVP level of computation.

```
30
symmetry c1
C       -1.132774000      2.052025000      0.011067000
N       -1.720735000      1.041775000      0.001697000
C       -2.399596000     -0.232056000     -0.004331000
C       -2.174632000     -0.886937000      1.358990000
C       -3.883726000      0.029565000     -0.256052000
C       -1.788367000     -1.080534000     -1.119532000
H       -2.579323000     -0.264949000      2.153810000
H       -2.679118000     -1.850523000      1.380307000
H       -1.113136000     -1.041000000      1.533454000
H       -4.301402000      0.658304000      0.526357000
H       -4.030134000      0.522207000     -1.214086000
H       -4.419115000     -0.917117000     -0.265619000
H       -0.728674000     -1.232186000     -0.933917000
H       -2.286312000     -2.047374000     -1.147484000
H       -1.915740000     -0.594990000     -2.084189000
O        1.186539000     -0.201309000      0.621179000
C        2.460377000     -0.015965000     -0.020183000
C        3.157998000     -1.362288000      0.082676000
C        3.246004000      1.064203000      0.716791000
C        2.240662000      0.376654000     -1.478644000
H        0.712658000      0.636216000      0.585757000
H        3.277605000     -1.644712000      1.126315000
H        2.570157000     -2.130159000     -0.415943000
H        4.140927000     -1.321166000     -0.382189000
H        2.718261000      2.016466000      0.672225000
H        3.371943000      0.789161000      1.761630000
H        4.230042000      1.202991000      0.271592000
H        3.190972000      0.506972000     -1.994128000
H        1.670509000     -0.393486000     -1.994299000
H        1.688347000      1.313461000     -1.542638000
```

Figure S21: Cartesian coordinates (XMOL format) of π-type t-BuOH$\cdots t$-BuNC computed at the B2PLYP-D3(BJ,abc)/ma-def2-QZVP level of computation.

2.4 Computed Properties

Table S8: Experimental and theoretical data on the 1:1 dimers of systems A, B, C and D. The zero point corrected energy difference between the σ- and π-type conformer is defined as $\Delta E_0 = E_{0,\pi} - E_{0,\sigma}$. The dimerization entropy contributions $T\Delta_{\text{dim}}S$ at room temperature ($T = 298.15\,\text{K}$), are obtained from harmonic calculations (B2PLYP-D3(BJ,abc)/ma-def2-QZVP). At room temperature, σ-complexes are seen to be entropically significantly favored over π-complexes in most cases, but this should not affect the findings in the supersonic jet too much, depending on the temperature at which the conformational equilibrium starts to freeze. If the latter is conservatively estimated at 100 K, the entropy effects do not exceed $1\,\text{kJ}\,\text{mol}^{-1}$, also because the rotational temperature of the generated complexes will be significantly lower. If observed, the experimental OHb-wavenumber $\tilde{\nu}_{\text{exp}}$ is given with the experimental uncertainty in parentheses. Scaled harmonic wavenumbers $\tilde{\nu}_{\text{calc}}$ and integrated absorption coefficients A_{calc} were obtained at the B3LYP-D3(BJ,abc)/def2-TZVP level of computation. ΔE_0 is calculated using the DLPNO-CCSD(T)//B2PLYP-D3(BJ,abc)/ma-def2-QZVP composite method.

	Dimer	Type	ΔE_0 / kJ mol^{-1}	$T\Delta_{\text{dim}}S$ / kJ mol^{-1}	$\tilde{\nu}_{\text{exp}}$ / cm^{-1}	$0.97\,\tilde{\nu}_{\text{calc}}$ / cm^{-1}	A_{calc} / km mol^{-1}
**A**	$H_2O\cdots t$-BuNC	σ	0.2	−29.8	3565(1)	3553	473
		π		−33.0	—	3632	58
**B**	t-BuOH$\cdots t$-BuNC	σ	−2.9	−43.1	3534(1)	3541	542
		π		−43.3	3596(1)	3635	106
**C**	$H_2O\cdots t$-BuCN	σ	−2.0	−30.8	3595(1)	3593	414
		π		−34.2	3613(1)	3621	80
**D**	t-BuOH$\cdots t$-BuCN	σ	−3.7	−42.2	—	3586	478
		π		−45.8	3590(1)	3623	133

Table S9: Scaled computed C≡N vibrational wavenumbers $\tilde{\nu}_{\text{calc}}$ and shifts $\Delta\tilde{\nu}_{\text{calc}} = \tilde{\nu}_{\text{dim}} - \tilde{\nu}_{\text{mono}}$ of computed dimer bands from the monomer band as well as the integrated absorption coefficients A_{calc} at the B3LYP-D3(BJ,abc)/def2-TZVP level of computation.

Species	$\tilde{\nu}_{\text{exp}}$ / cm^{-1}	$0.96\,\tilde{\nu}_{\text{calc}}$ / cm^{-1}	$\Delta\tilde{\nu}_{\text{calc}}$ / cm^{-1}	A_{calc} / km mol^{-1}
t-BuNC	2141(1)	2130	—	141
t-BuNC$\cdots t$-BuNC	2148(1)	2143	+13	223
$H_2O\cdots t$-BuNC (σ)	2160(1)	2156	+26	130
$H_2O\cdots t$-BuNC (π)	—	2135	+5	105
t-BuCN	2246(1)	2250	—	12
t-BuCN$\cdots t$-BuCN	2244(1)	2247	−3	54
$H_2O\cdots t$-BuCN (σ)	—	2261	+11	27
$H_2O\cdots t$-BuCN (π)	—	2241	−9	25

Table S10: Experimental and theoretical wavenumber shifts relative to the monomer $\Delta\tilde{\nu} = \tilde{\nu}_{\text{mono}} - \tilde{\nu}_{\text{dim}}$ for the 1:1 dimers of systems A, B, C and D in the O–H and C≡N vibrations. For H_2O, the symmetric stretching fundamental at 3657 cm^{-1} [32] (exp.) / 3782 cm^{-1} (calc.) and for *t*-BuOH, 3642 cm^{-1} [33] (exp.) / 3785 cm^{-1} (calc.) are taken as the reference monomer wavenumbers for the OHb downshift. The predicted values are taken from B3LYP-D3(BJ,abc)/def2-TZVP computations. All values are given in cm^{-1}.

	Dimer	Type	$\Delta\tilde{\nu}_{\text{exp}}$(OHb)	$\Delta\tilde{\nu}_{\text{calc}}$(OHb)	$\Delta\tilde{\nu}_{\text{exp}}$(C≡N)	$\Delta\tilde{\nu}_{\text{calc}}$(C≡N)
**A**	$H_2O\cdots$ *t*-BuNC	σ	−92(1)	−119	+18(2)	+26
		π	—	−37	—	+5
**B**	*t*-BuOH$\cdots$ *t*-BuNC	σ	−108(1)	−135	—	+26
		π	−46(1)	−37	—	+3
**C**	$H_2O\cdots$ *t*-BuCN	σ	−62(1)	−78	—	+11
		π	−44(1)	−49	—	−10
**D**	*t*-BuOH$\cdots$ *t*-BuCN	σ	—	−88	—	+10
		π	−52(1)	−50	—	−10

Table S11: Computed interaction energies ΔE_{int} and dissociation energies with (D_0) and without (D_e) zero-point correction. D_0 was computed using the DLPNO-CCSD(T)//B2PLYP-D3(BJ,abc)/ma-def2-QZVP composite method, while ΔE_{int} and D_e were calculated at the DLPNO-CCSD(T)/ma-def2-QZVP level of computation without zero-point correction. Geometries were obtained from a B2PLYP-D3(BJ,abc)/ma-def2-QZVP geometry optimization.

	Dimer	Type	$-\Delta E_{\text{int}}$ / kJ mol^{-1}	D_e / kJ mol^{-1}	D_0 / kJ mol^{-1}
**A**	$H_2O\cdots$ *t*-BuNC	σ	21.4	21.0	15.4
		π	20.6	20.4	15.2
**B**	*t*-BuOH$\cdots$ *t*-BuNC	σ	22.6	22.3	18.6
		π	24.1	23.9	21.5
**C**	$H_2O\cdots$ *t*-BuCN	σ	21.3	21.1	15.1
		π	23.5	23.1	17.1
**D**	*t*-BuOH$\cdots$ *t*-BuCN	σ	22.9	22.7	19.2
		π	26.8	26.5	22.9

Table S12: Electrostatic properties of *t*-BuNC, *t*-BuCN, H_2O and *t*-BuOH, computed at B3LYP/def2-TZVP level of computation. All components of the dipole vector $\vec{\mu}$ and its magnitude are given in D, all independent components of the traceless quadrupole tensor $\boldsymbol{\Theta}$ with respect to the center of mass (COM) are given in D Å and the eigenvalues of the polarizability volume tensor $\boldsymbol{\alpha'}$ as well as the isotropic polarizability volume trace($\boldsymbol{\alpha'}$)/3 are given in Å^3. The *z*-axis is defined along the C_n axis if present. For H_2O, the molecular plane is defined as *yz* and for *t*-BuOH, *yz* is the plane of reflection symmetry.

	t-BuNC	*t*-BuCN	H_2O	*t*-BuOH
Point Group	C_{3v}	C_{3v}	C_{2v}	C_s
$\lvert\mu_x\rvert$ / D	0.00	0.00	0.00	0.00
$\lvert\mu_y\rvert$ / D	0.00	0.00	0.00	1.33
$\lvert\mu_z\rvert$ / D	4.08	4.12	2.06	0.83
$\lvert\vec{\mu}\rvert$ / D	4.08	4.12	2.06	1.56
Θ_{xx} / D Å	3.5	3.4	−1.6	−0.3
Θ_{yy} / D Å	3.5	3.4	1.7	−2.4
Θ_{xy} / D Å	0.0	0.0	0.0	0.0
Θ_{xz} / D Å	0.0	0.0	0.0	0.0
Θ_{yz} / D Å	0.0	0.0	0.0	1.7
α'_{xx} / Å^3	9.0	8.7	0.8	8.2
α'_{yy} / Å^3	9.0	8.7	1.2	7.9
α'_{zz} / Å^3	11.1	10.7	1.0	8.4
$\bar{\alpha}'$ / Å^3	9.7	9.4	1.0	8.2

2.5 Experimental-Theoretical Abundances

2.5.1 OH region

In order to estimate the relative conformer abundances χ of the competing π and σ type structures, experimental band integrals I_{exp} and computationally predicted intensities A_{calc} are combined.

The spectral bands are integrated by using a Monte-Carlo approach based on noise characteristics.[34] Analogous to Ref. [35], we identify the average full-width-half-maximum (FWHM$=$ 5 cm^{-1}) of the experimental bands to define two symmetric integration windows around the band center B as $B \pm$ (FWHM $+$ 2 cm^{-1}) and $B \pm$ (FWHM $+$ 4 cm^{-1}), where the FWHM value is stochastically varied by 2 cm^{-1}. This yields two integrals I_1 and I_2 which determine the mean absorbance integral as $I_{exp} = 0.5 \cdot (I_1 + I_2)$. The corresponding uncertainty is calculated as $\Delta I_{exp}{}^{\mathrm{I}} = 0.5 \cdot (I_1 - I_2)$. As the integral uncertainty mainly depends on spectral noise it should remain approximately constant across all four spectra. Thus, for all the following calcula-

tions, the largest uncertainty is taken as a conservative uncertainty estimate for all integrals ($\Delta I_{\text{exp}}{}^{\text{II}} = 0.22\,\mu\text{m}^{-1}$).
If both conformers are observed (systems B and C), maximal and minimal experimental intensity ratios are computed as $(I_{\text{exp}}{}^{\pi} + \Delta I_{\text{exp}}{}^{\pi})/(I_{\text{exp}}{}^{\sigma} - \Delta I_{\text{exp}}{}^{\sigma})$ and $(I_{\text{exp}}{}^{\pi} - \Delta I_{\text{exp}}{}^{\pi})/(I_{\text{exp}}{}^{\sigma} + \Delta I_{\text{exp}}{}^{\sigma})$, respectively. This is divided by the theoretical intensity ratio $A_{\text{calc}}{}^{\pi}/A_{\text{calc}}{}^{\sigma}$ (see Table S8) to obtain corrected conformer ratios $R_{\pm}$ (Equation S1).
As the π-type conformer is not observed for system A, its spectral band integral amounts to $I_{\text{exp}}{}^{\pi} = 0$. Thus, the maximal non-corrected integral ratio can be written as $\Delta I_{\text{exp}}{}^{\sigma}/(I_{\text{exp}}{}^{\sigma} - \Delta I_{\text{exp}}{}^{\sigma})$. As negative intensities are not meaningful in this context, the minimal non-corrected integral ratio amounts to $0/(I_{\text{exp}}{}^{\sigma} + \Delta I_{\text{exp}}{}^{\sigma}) = 0$. For D, where only the π type conformer is experimentally observed, the non-corrected maximal and minimal intensity ratios are given as $(I_{\text{exp}}{}^{\pi} + \Delta I_{\text{exp}}{}^{\pi})/0 \to \infty$ and $(I_{\text{exp}}{}^{\pi} - \Delta I_{\text{exp}}{}^{\pi})/\Delta I_{\text{exp}}{}^{\pi}$, respectively. For these spectral analyses, we assume that no dimer bands overlap. The full equations for the theoretically corrected $R_{\pm}$ values are shown in Equation S1.
The abundances $\chi_{\pm}$ are calculated according to Equation S2 and Equation S3.)

$$R_{\pm} = \begin{cases} \dfrac{I_{\text{exp}}{}^{\pi} \pm \Delta I_{\text{exp}}{}^{\pi}}{I_{\text{exp}}{}^{\sigma} \mp \Delta I_{\text{exp}}{}^{\sigma}} \cdot \dfrac{A_{\text{calc}}{}^{\sigma}}{A_{\text{calc}}{}^{\pi}}, & \text{if both } \sigma \text{ and } \pi \text{ observed,} \\ \dfrac{1/2\left(\Delta I_{\text{exp}}{}^{\sigma} \pm \Delta I_{\text{exp}}{}^{\sigma}\right)}{I_{\text{exp}}{}^{\sigma} \mp \Delta I_{\text{exp}}{}^{\sigma}} \cdot \dfrac{A_{\text{calc}}{}^{\sigma}}{A_{\text{calc}}{}^{\pi}}, & \text{if only } \sigma \text{ observed,} \\ \dfrac{I_{\text{exp}}{}^{\pi} \pm \Delta I_{\text{exp}}{}^{\pi}}{1/2\left(\Delta I_{\text{exp}}{}^{\pi} \mp \Delta I_{\text{exp}}{}^{\pi}\right)} \cdot \dfrac{A_{\text{calc}}{}^{\sigma}}{A_{\text{calc}}{}^{\pi}}, & \text{if only } \pi \text{ observed.} \end{cases} \tag{S1}$$

$$\chi_{\pm}{}^{\sigma} = \frac{1}{R_{\pm} + 1} \tag{S2}$$

$$\chi_{\pm}{}^{\pi} = \frac{R_{\pm}}{R_{\pm} + 1} \tag{S3}$$

Table S13: Experimentally determined decadic OHb absorbance integrals $I_{exp} = \int_{conf} A_{10}\, d\tilde{\nu}$ and their uncertainties $\Delta I_{exp}{}^{I/II}$ as well as the maximum and minimum theoretically corrected ratios $R_{\pm}$ (Equation S1) and the corresponding abundances $\chi_{\pm}$ according to Equation S2 and Equation S3. For harmonically assessed theoretical cross sections, no uncertainty was assumed in these estimates.

	Dimer	Type	I_{exp} / µm^{-1}	$\Delta I_{exp}{}^{I}$ / µm^{-1}	$\Delta I_{exp}{}^{II}$ / µm^{-1}	R_+	R_-	χ_+ / %	χ_- / %
**A**	$H_2O\cdots t$-BuNC	σ	3.38	0.13	0.22	0.57	0	64	100
		π	—	—	—			36	0
**B**	t-BuOH$\cdots t$-BuNC	σ	1.53	0.16	0.22	11	6.8	8	13
		π	2.55	0.02	0.22			92	87
**C**	$H_2O\cdots t$-BuCN	σ	0.67	0.07	0.22	12	3.7	7	21
		π	0.86	0.01	0.22			93	79
**D**	t-BuOH$\cdots t$-BuCN	σ	—	—	—	∞	64	0	2
		π	4.15	0.22	0.22			100	98

2.5.2 C≡N region

I: Relative abundances of monomers and homodimers

Since spectral overlap renders the integration method described in subsubsection 2.5.1 inapplicable, another approach is employed to assume the spectral absorption integrals of the partially overlapping monomer and homodimer signals in *t*-BuNC spectra.

For this, a Gaussian lineshape $G(\tilde{\nu})$, given by Equation S4, was assumed for both signals and fit manually to the spectral bands.

$$G(\tilde{\nu}) = a \cdot \exp\left(-\frac{1}{2}\left(\frac{\tilde{\nu}-\tilde{\nu}_0}{b}\right)^2\right) \tag{S4}$$

From this, the absorption integral is computed using the analytic Gaussian solution,

$$\int_{-\infty}^{\infty} G(\tilde{\nu})\, d\tilde{\nu} = a \cdot b \int_{-\infty}^{\infty} \exp\left(-\frac{x^2}{2}\right) dx = \sqrt{2\pi} \cdot a \cdot b, \tag{S5}$$

which depends linearly on a and b. The fitted curves are shown in Figure S22 and Figure S23.

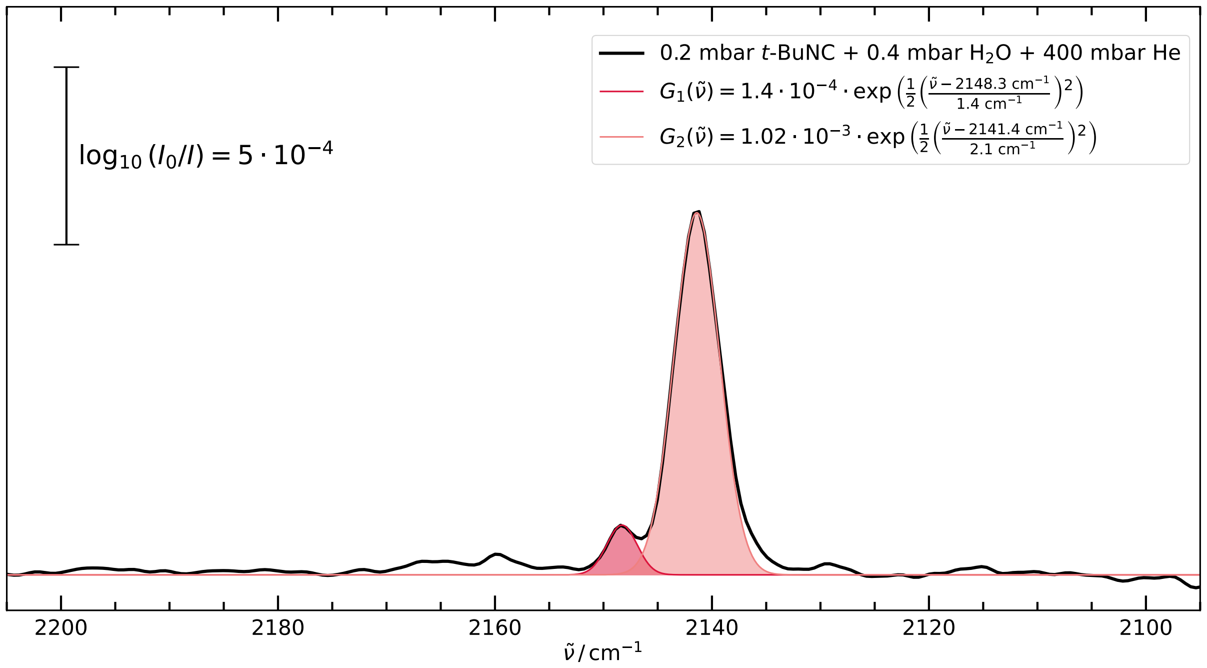

Figure S22: Manually fitted Gaussian curves for monomer and homodimer bands of *tert*-butyl isocyanide with He as the carrier gas.

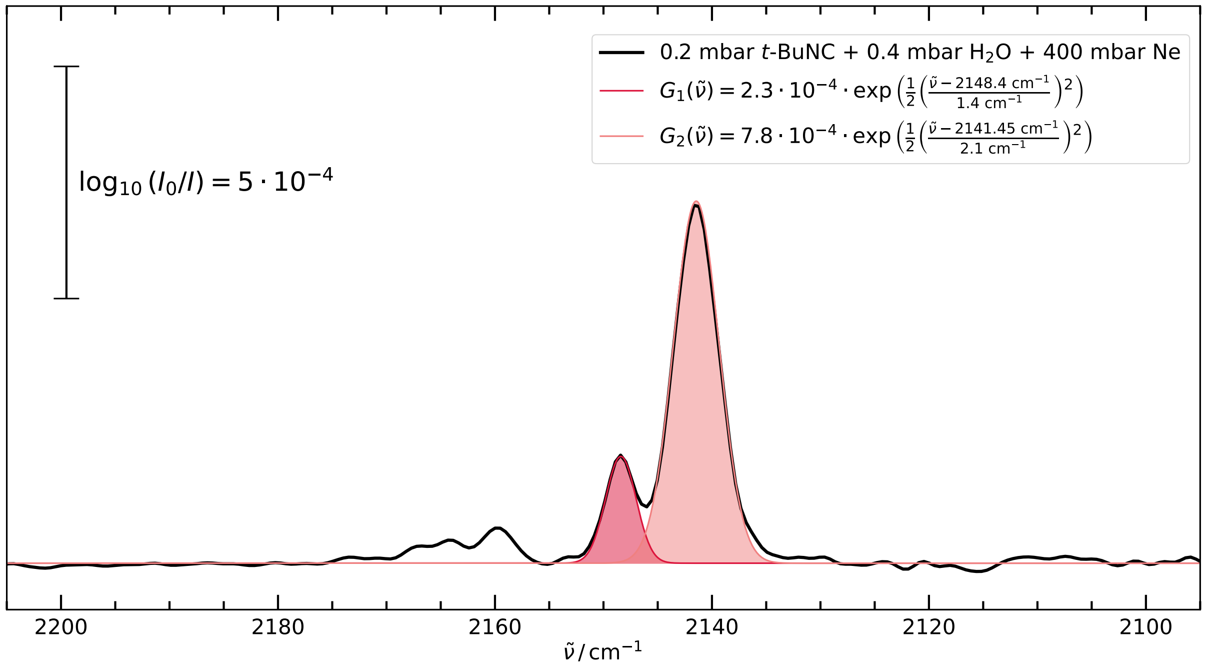

Figure S23: Manually fitted Gaussian curves for monomer and homodimer bands of *tert*-butyl isocyanide with Ne as the carrier gas.

The optimized parameters and the resulting integrals are summarized in Table S14.

Table S14: Overview of the optimized parameters a, $\tilde{\nu}$ and b of the Gaussian lineshape as given by Equation S4. Additionally, the analytical integral values calculated by Equation S5 are summarized, from which monomer and homodimer shares from $(t\text{-BuNC})_n$, $n = 1, 2$ species χ are calculated.

Carrier Gas	He		Ne	
Species	t-BuNC	$(t\text{-BuNC})_2$	t-BuNC	$(t\text{-BuNC})_2$
$a\,/\,10^{-3}$	1.02	0.14	0.78	0.23
$\tilde{\nu}\,/\,\mathrm{cm}^{-1}$	2141.4	2148.3	2141.45	2148.4
$b\,/\,\mathrm{cm}^{-1}$	2.1	1.4	2.1	1.4
$\int_{-\infty}^{\infty} G(\tilde{\nu})\,\mathrm{d}\tilde{\nu}\,/\,10^{-3}\,\mathrm{cm}^{-1}$	5.37	0.49	4.11	0.81
$\chi\,/\,\%$	94.5	5.5	89	11

After applying harmonically approximated IR cross-sections, the abundance of homodimer species from all $(t\text{-BuNC})_n$, $n = 1, 2$ species in the expansion can be estimated with Equation S3. Using this analysis, the homodimer share amounts to 5.5 % in He or 11 % in Ne. Since this analysis is not used for quantitatively accurate but rather for instructive purposes, no uncertainties are estimated.

II: Benchmarking computed intensity ratios of different modes within a cluster

In cases of sufficiently separated cluster bands, the comparison of C≡N and O−H stretching vibration bands can be used to benchmark computed intensity ratios of different modes within a cluster. For $H_2O\cdots t$-BuNC, the σ-type heterodimer has been observed in both spectral regions. While the integration of the OHb band of the σ-type complex is straightforward (subsubsection 2.5.1), its NC stretching band overlaps with other bands. Thus, only minimum and maximum intensities can be determined. The integration results are shown in Table S15. From these, the minimum and maximum intensity ratios $R_\pm$ of the OHb and NC bands are derived using Equation S6.

$$R_\pm = \frac{\bar{I}_{\mathrm{NC}} \pm \Delta \bar{I}_{\mathrm{NC}}}{I_{\mathrm{OHb}} \mp \Delta I_{\mathrm{OHb}}} \tag{S6}$$

The extreme values are used to compute the mean $\bar{R}$ according to Equation S7. The uncertainty range ΔR (Equation S8) is set in a way that it includes both R_+ and R_-.

$$\bar{R} = \frac{R_+ + R_-}{2} \tag{S7}$$

$$\Delta\bar{R} = \frac{R_{+} - R_{-}}{2} \tag{S8}$$

Table S15: Experimentally determined decadic OHb and NC absorbance integrals $I_{\text{exp}}{}^{\sigma}$ of σ-type $H_2O\cdots t$-BuNC. For the spectral integration the '20250926-abcd-tert-Butylisocyanide-0.200mbar-H2O-0.400mbar-Ne-400mbar-p_s-0,4barpm-Av.800' spectrum was used (see Table S1). R was determined using Equation S6 and Equation S7. The integrated absorption coefficients (B3LYP/def2-TZVP level of theory) are given as $A_{\text{calc}}{}^{\sigma}$.

t-BuNC–H_2O	OHb	NC	R
$I_{\text{exp}}{}^{\sigma}$ / µm^{-1}	6.05 ± 0.08	$I_{-} = 1.02 \pm 0.04$<br>$I_{+} = 3.22 \pm 0.20$<br>$\bar{I} = 2.12 \pm 1.10$	$R_{-} = 0.16$<br>$R_{+} = 0.54$<br>$\bar{R} = 0.35 \pm 0.19$
$A_{\text{calc}}{}^{\sigma}$ / km mol^{-1}	473	130	0.27

3 Non-Covalent Interaction Plots

Non-covalent interaction (NCI) plots[36] were generated using electron densities (.gbw) from Orca B2PLYP-D3(BJ,abc)/ma-def2-QZVP computations. These were then processed using MultiWFN[37] (v. 3.8) and visualized with VMD[38] (v. 1.9.3).

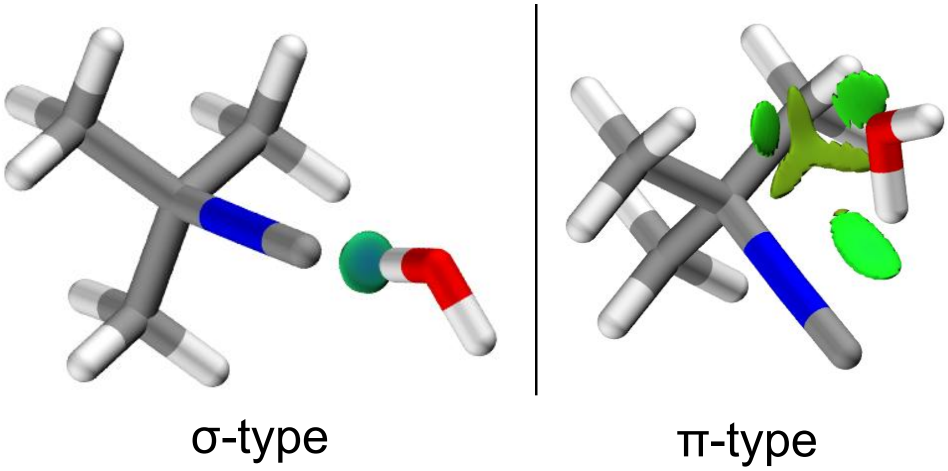

Figure S24: NCI plots of $H_2O\cdots t$-BuNC dimers (**A**) using the electron density obtained from a computation at the B2PLYP-D3(BJ,abc)/ma-def2-QZVP level of computation. The isosurface is plotted at $s = 0.5$.

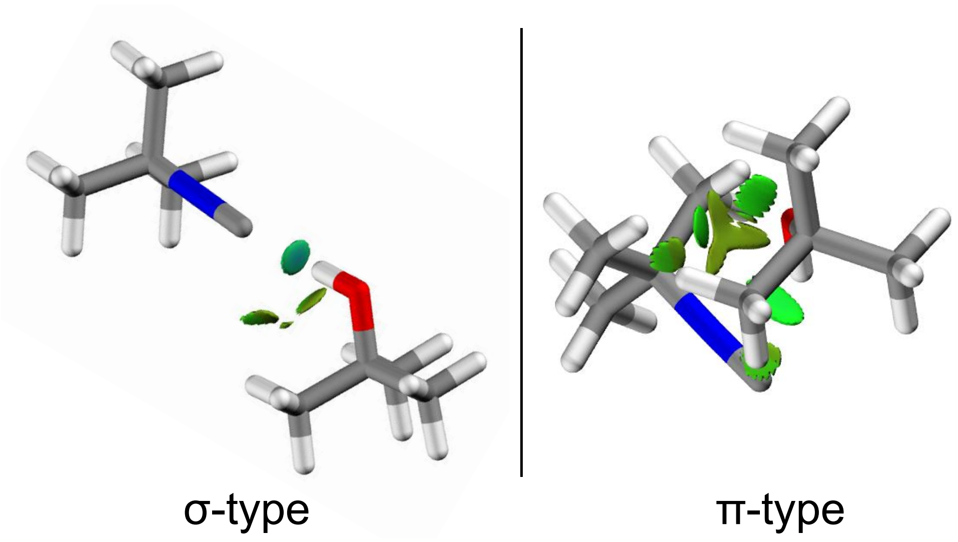

Figure S25: NCI plots of t-BuOH$\cdots t$-BuNC dimers (**B**) using the electron density obtained from a computation at the B2PLYP-D3(BJ,abc)/ma-def2-QZVP level of computation. The isosurface is plotted at $s = 0.5$.

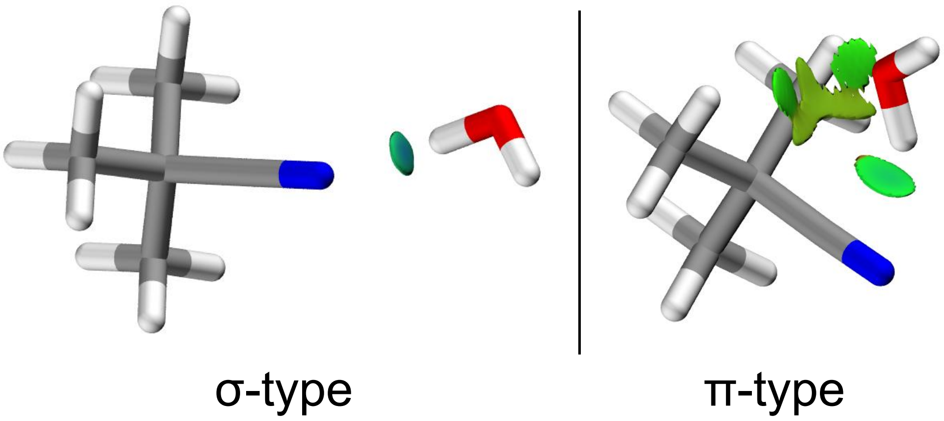

Figure S26: NCI plots of $H_2O\cdots t$-BuCN dimers (**C**) using the electron density obtained from a computation at the B2PLYP-D3(BJ,abc)/ma-def2-QZVP level of computation. The isosurface is plotted at $s = 0.5$.

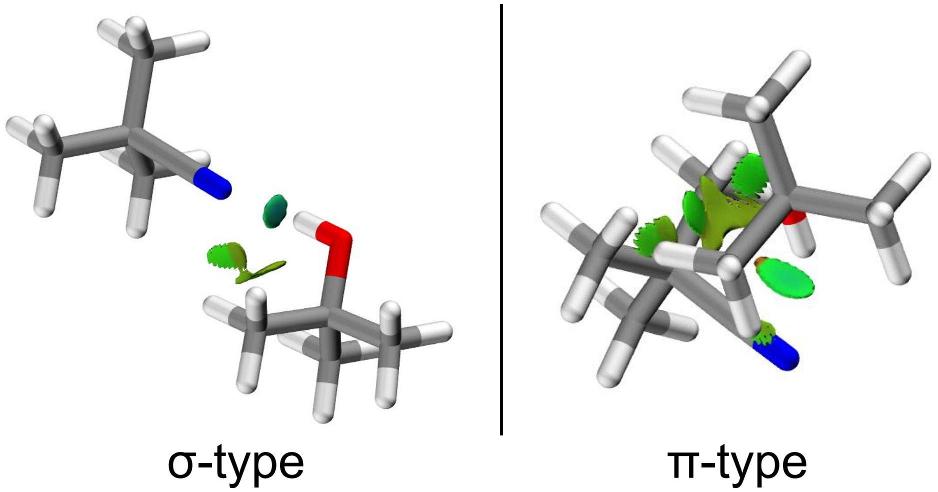

Figure S27: NCI plots of t-BuOH$\cdots$$t$-BuCN dimers (**D**) using the electron density obtained from a computation at the B2PLYP-D3(BJ,abc)/ma-def2-QZVP level of computation. The isosurface is plotted at $s = 0.5$.

4 Other Figures

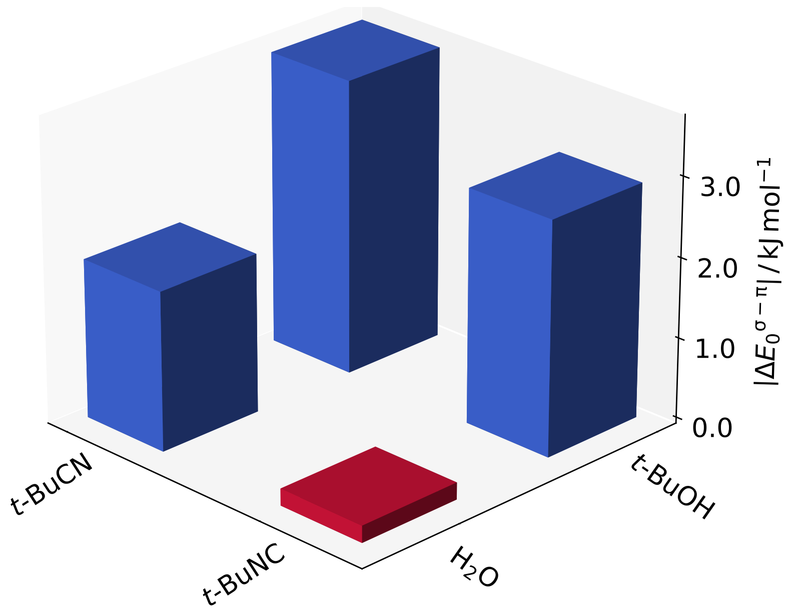

Figure S28: Computed zero-point corrected stability differences between σ- (linear-) and π-(side-)type conformers using the DLPNO-CCSD(T)//B2PLYP-D3(BJ,abc)/ma-def2-QZVP composite method. Blue bars mark a stability edge in favor of the side-type conformer while the red bar encodes a stability advantage for the linear-type conformer. The raw D_0 values are summarized in Table S11.

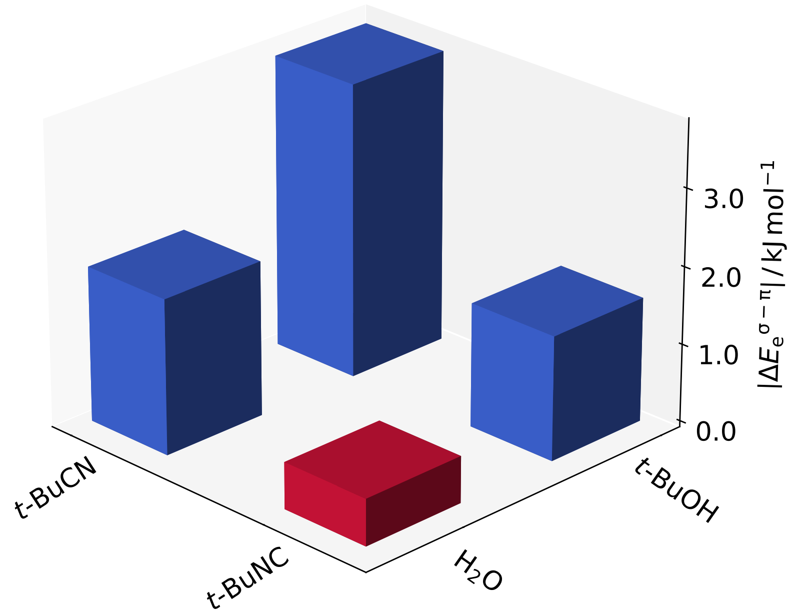

Figure S29: Computed electronic stability differences between σ- and π-type conformers using the DLPNO-CCSD(T)//B2PLYP-D3(BJ,abc)/ma-def2-QZVP composite method. Blue bars mark a stability edge in favor of the side-type conformer while the red bar encodes a stability advantage for the linear-type conformer. The raw D_e values are summarized in Table S11.

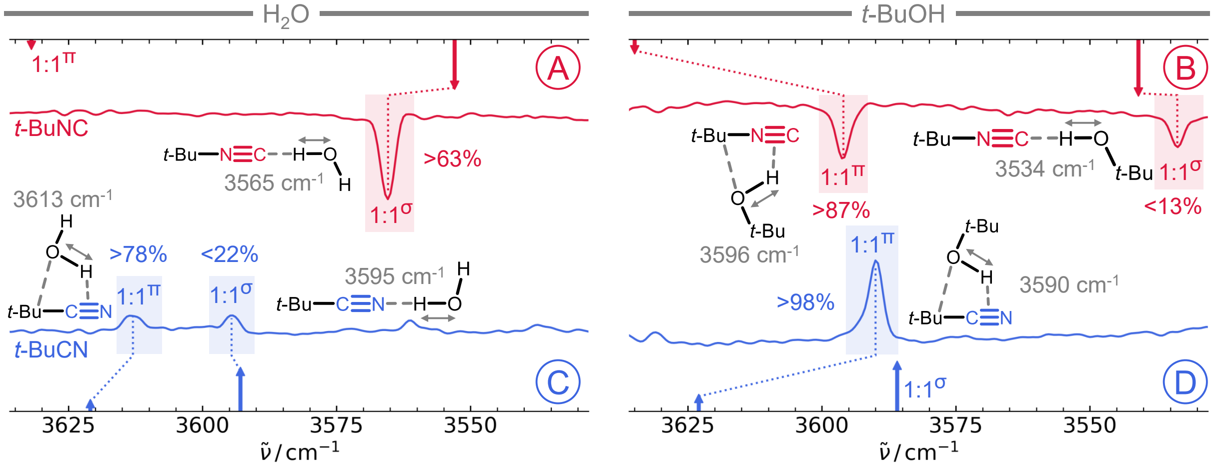

Figure S30: Jet cooled FTIR spectra of t-BuNC with H_2O (A) or t-BuOH (B), and of t-BuCN with H_2O (C) or t-BuOH (D), plotted similar to Figure 3 (main text) as absolute wavenumbers. All measurements were performed with a partial pressure of 0.4 mbar (H_2O, t-BuOH) or 0.2 mbar (t-BuCN, t-BuNC) in 400 mbar He. If possible, scaled harmonic wavenumbers (arrows, B3LYP-D3(BJ,abc)/def2-TZVP, uniform scaling factor 0.97, see Table S8) of the 1:1-complexes are assigned to experimental bands (see Table S5). The arrow size represents the integrated absorption coefficient. The comparison between Figure 3 and Figure S30 suggests that monomer wavenumber scaling in combination with unscaled harmonic complexation shifts is a better strategy to remove the errors inherent in a harmonic DFT approach than uniform scaling of predicted harmonic wavenumbers, when different hydrogen bond donors are involved.

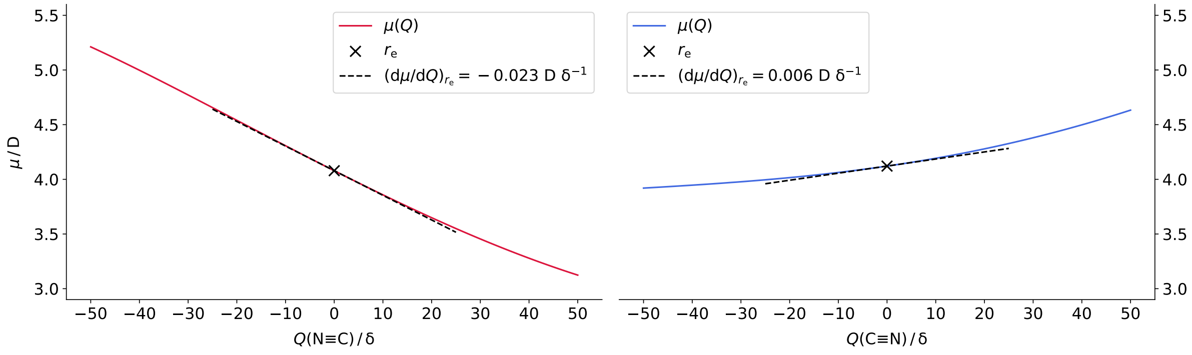

Figure S31: Dipole curves with respect to the N≡C and C≡N stretching normal mode coordinates of t-BuNC and t-BuCN, respectively. Both curves were created using data from an ORCA normal mode coordinate scan at the B3LYP/def2-TZVP level of computation with ± 50 displacements from the equilibrium geometry with stepsize $\delta = 0.1$ of the (unitless and normalized) normal mode vector.

References

[1] H. C. Gottschalk, PhD thesis, Niedersächsische Staats-und Universitätsbibliothek Göttingen, **2020**, DOI 10.53846/goediss-8308.

[2] H. C. Gottschalk, T. L. Fischer, V. Meyer, R. Hildebrandt, U. Schmitt, M. A. Suhm, *Instruments* **2021**, *5*, 12.

[3] E. Lwin, M. J. Gölz, N. O. B. Lüttschwager, M. A. Suhm, S. Käser, V. Andreichev, M. A. Brandes, M. Meuwly, *Phys. Chem. Chem. Phys.* **2025**, *27*, 17692–17703.

[4] J. D. Hunter, *Comput. Sci. Eng.* **2007**, *9*, 90–95.

[5] G. Van Rossum, F. L. Drake, *Python 3 Reference Manual*, CreateSpace, Scotts Valley, CA, **2009**.

[6] C. R. Harris, K. J. Millman, S. J. van der Walt, R. Gommers, P. Virtanen, D. Cournapeau, E. Wieser, J. Taylor, S. Berg, N. J. Smith, R. Kern, M. Picus, S. Hoyer, M. H. van Kerkwijk, M. Brett, A. Haldane, J. F. del Río, M. Wiebe, P. Peterson, P. Gérard-Marchant, K. Sheppard, T. Reddy, W. Weckesser, H. Abbasi, C. Gohlke, T. E. Oliphant, *Nature* **2020**, *585*, 357–362.

[7] J. Duran, BrukerOpus: A Python library for reading Bruker OPUS files, Accessed: 2025-08-01, **2021**.

[8] J. H. Lambert, *Photometria sive de mensura et gradibus luminis, colorum et umbrae*, sumptibus vidvae E. Klett, typis CP Detleffsen, Augsburg, **1760**.

[9] C. Camy-Peyret, J. Flaud, G. Guelachvili, C. Amiot, *Mol. Phys.* **1973**, *26*, 825–855.

[10] F. Neese, *Wiley Interdiscip. Rev. Comput. Mol. Sci.* **2012**, *2*, 73–78.

[11] F. Neese, *Wiley Interdiscip. Rev. Comput. Mol. Sci.* **2022**, *12*, e1606.

[12] S. Grimme, *J. Chem. Theory Comput.* **2019**, *15*, 2847–2862.

[13] P. Pracht, F. Bohle, S. Grimme, *Phys. Chem. Chem. Phys.* **2020**, *22*, 7169–7192.

[14] S. Grimme, C. Bannwarth, P. Shushkov, *J. Chem. Theory Comput.* **2017**, *13*, 1989–2009.

[15] C. Bannwarth, S. Ehlert, S. Grimme, *J. Chem. Theory Comput.* **2019**, *15*, 1652–1671.

[16] A. D. Becke, *Phys. Rev. A* **1988**, *38*, 3098.

[17] C. Lee, W. Yang, R. G. Parr, *Phys. Rev. B* **1988**, *37*, 785.

[18] B. Miehlich, A. Savin, H. Stoll, H. Preuss, *Chem. Phys. Lett.* **1989**, *157*, 200–206.

[19] A. D. Becke, *J. Chem. Phys.* **1993**, *98*, 5648–5652.

[20] S. Grimme, J. Antony, S. Ehrlich, H. Krieg, *J. Chem. Phys.* **2010**, *132*, 154104.

[21] A. D. Becke, E. R. Johnson, *J. Chem. Phys.* **2005**, *123*, 154101.

[22] E. R. Johnson, A. D. Becke, *J. Chem. Phys.* **2005**, *123*, 024101.

[23] E. R. Johnson, A. D. Becke, *J. Chem. Phys.* **2006**, *124*, 174104.

[24] S. Grimme, S. Ehrlich, L. Goerigk, *J. Comput. Chem.* **2011**, *32*, 1456–1465.

[25] F. Weigend, R. Ahlrichs, *Phys. Chem. Chem. Phys.* **2005**, *7*, 3297–3305.

[26] S. Grimme, *J. Chem. Phys.* **2006**, *124*, 034108.

[27] T. Schwabe, S. Grimme, *Phys. Chem. Chem. Phys.* **2007**, *9*, 3397–3406.

[28] J. Zheng, X. Xu, D. G. Truhlar, *Theor. Chem. Acc.* **2011**, *128*, 295–305.

[29] J. Čížek, *J. Chem. Phys.* **1966**, *45*, 4256–4266.

[30] J. Čížek, *Adv. Chem. Phys.* **1969**, *14*, 35–89.

[31] Y. Guo, C. Riplinger, U. Becker, D. G. Liakos, Y. Minenkov, L. Cavallo, F. Neese, *J. Chem. Phys.* **2018**, *148*, 011101.

[32] K. E. Otto, Z. Xue, P. Zielke, M. A. Suhm, *Phys. Chem. Chem. Phys.* **2014**, *16*, 9849–9858.

[33] D. Bernhard, F. Dietrich, M. Fatima, C. Perez, A. Poblotzki, G. Jansen, M. A. Suhm, M. Schnell, M. Gerhards, *Phys. Chem. Chem. Phys.* **2017**, *19*, 18076–18088.

[34] N. Lüttschwager, *J. Open Source Softw.* **2021**, *6*, 3526.

[35] M. Bödecker, D. Mihrin, M. A. Suhm, R. Wugt Larsen, *J. Phys. Chem. A* **2024**, *128*, 7124–7136.

[36] E. R. Johnson, S. Keinan, P. Mori-Sánchez, J. Contreras-García, A. J. Cohen, W. Yang, *J. Am. Chem. Soc.* **2010**, *132*, 6498–6506.

[37] T. Lu, F. Chen, *J. Comput. Chem* **2012**, *33*, 580–592.

[38] W. Humphrey, A. Dalke, K. Schulten, *J. Mol. Graph.* **1996**, *14*, 33–38.
